# Contextualizing biological heterogeneity in brain structural normativity assessments across the aging continuum with the nearest neighbor normativity framework

**DOI:** 10.1038/s43856-026-01903-0

**Published:** 2026-11-13

**Authors:** Ramona Leenings, Nils R. Winter, Jan Ernsting, Maximilian Konowski, Vincent Holstein, Susanne Meinert, Jennifer Spanagel, Carlotta Barkhau, Lukas Fisch, Janik Goltermann, Malte F. Gerdes, Dominik Grotegerd, Elisabeth J. Leehr, Annette Peters, Lilian Krist, Stefan N. Willich, Tobias Pischon, Henry Völzke, Johannes Haubold, Hans-Ulrich Kauczor, Thoralf Niendorf, Maike Richter, Udo Dannlowski, Klaus Berger, Xiaoyi Jiang, James Cole, Nils Opel, Tim Hahn

**Affiliations:** 1 Faculty of Mathematics and Computer Science, University of Münster, Münster, Germany; 2 Department of Psychiatry and Neurosciences, Charité - Universitätsmedizin Berlin, Berlin, Germany; 3 University of Münster, Institute for Translational Psychiatry, Münster, Germany; 4 Department of Neurology, Kepler University Hospital, Johannes Kepler University Linz, Linz, Austria; 5 Institute for Geoinformatics, University of Münster, Münster, Germany; 6 McLean Hospital, Belmont, MA USA; 7 Department of Psychiatry, Harvard Medical School, Boston, MA USA; 8 Stanley Center for Psychiatric Research, Broad Institute of MIT and Harvard, Cambridge, MA USA; 9 Institut for Translational Neuroscience, University of Münster, Münster, Germany; 10 Institute of Epidemiology, Helmholtz Zentrum M¨unchen, German Research Center for Environmental Health (GmbH), Neuherberg, Germany; 11 Chair of Epidemiology, Institute for Medical Information Processing, Biometry and Epidemiology, Ludwig-Maximilians-Universitat Munchen, Munich, Germany; 12 German Center for Diabetes Research (DZD e.V.), Neuherberg, Germany; 13 German Center for Mental Health (DZPG), partner site Munich, Munich, Germany; 14 Institute of Social Medicine, Epidemiology and Health Economics, Charité - Universitätsmedizin Berlin, Berlin, Germany; 15 Molecular Epidemiology Research Group, Max Delbrück Center for Molecular Medicine in the Helmholtz Association (MDC), Berlin, Germany; 16 German Centre for Cardiovascular Research (DZHK), Greifswald, Germany; 17 Institute for Community Medicine, University Medicine Greifswald, Greifswald, Germany; 18 Institute of Diagnostic and Interventional Radiology and Neuroradiology, University Hospital Essen, Essen, Germany; 19 Institute for Artificial Intelligence in Medicine (IKIM), University Hospital Essen, Essen, Germany; 20 Diagnostic and Interventional Radiology, University Hospital Heidelberg, Heidelberg, Germany; 21 Berlin Ultrahigh Field Facility, Max Delbrück Center for Molecular Medicine in the Helmholtz Association (MDC), Berlin, Germany; 22 Site Berlin-Potsdam, German Center for Mental Health (DZPG), Berlin, Germany; 23 Department of Psychiatry, Medical School and University Medical Center OWL, Protestant Hospital of the Bethel Foundation, Bielefeld University, Bielefeld, Germany; 24 Site Jena Magdeburg Halle, German Center for Mental Health (DZPG), Jena, Germany; 25 Site Jena Magdeburg Halle, Center for Intervention and Research on Adaptive and Maladaptive Brain Circuits Underlying Mental Health (C-I-R-C), Jena, Germany; 26 Institute of Epidemiology and Social Medicine, University of Münster, Münster, Germany; 27 UCL Hawkes Institute, Department of Computer Science, University College London, London, UK; 28 Dementia Research Centre, Queen Square Institute of Neurology, University College London, London, UK; 29 University of California, San Francisco, San Francisco, CA USA; 30 Northern California Institute for Research and Education, San Francisco, CA USA; 31 University of Southern California, Los Angeles, CA USA; 32 Mayo Clinic, Rochester, MN USA; 33 University of California, Berkeley, Berkeley, CA USA; 34 Fordham University, New York, NY USA; 35 Mount Sinai Medical Center, New York, NY USA; 36 University of Wisconsin, Madison, USA; 37 University of Pennsylvania, Philadelphia, PA USA; 38 University of California, Los Angeles, Los Angeles, CA USA; 39 University of California, Davis, CA USA; 40 Boston University, Boston, CA USA; 41 Harvard University, Cambridge, MA USA; 42 Indiana University, Indianapolis, IN USA; 43 Washington University, St. Louis, MO USA; 44 Eisai, Nutley, NJ USA; 45 University of Washington, Seattle, MA USA; 46 University College London, London, UK; 47 University of Michigan, Ann Arbor, MI USA; 48 University of Pittsburgh, Pittsburgh, PA USA; 49 Duke University, Durham, NC USA; 50 University of California, Irvine, Irvine, CA USA; 51 Khachaturian, Radebaugh & Associates (KRA), Inc., Potomac, MD USA; 52 Prevent Alzheimer’s Disease, Rockville, MD USA; 53 General Electric, Boston, MA USA; 54 University of Connecticut, Storrs, CT USA; 55 National Institute on Aging, Bethesda, MD USA; 56 National Institutes of Health, Bethesda, MD USA; 57 Alzheimer’s Association, Chicago, IL USA; 58 National Institute of Mental Health, Bethesda, MD USA; 59 Rush University Medical Center, Chicago, IL USA; 60 University of California, San Diego, San Diego, CA USA; 61 Denali Therapeutics, South San Francisco, CA USA; 62 Massachusetts General Hospital, Boston, MA USA; 63 Biogen, Cambridge, MA USA; 64 Oregon Health and Science University, Portland, OR USA; 65 Baylor College of Medicine, Houston, TX USA; 66 Columbia University Medical Center, New York, NY USA; 67 University of Alabama at Birmingham, Birmingham, AL USA; 68 Wien Center, Miami, FL USA; 69 Johns Hopkins University, Baltimore, MD USA; 70 New York University, New York, NY USA; 71 University of Kentucky, Lexington, KY USA; 72 University of Rochester Medical Center, Rochester, NY USA; 73 University of Texas Southwestern Medical School, Dallas, TX USA; 74 Emory University, Atlanta, GA USA; 75 University of Kansas Medical Center, Kansas City, KS USA; 76 Mayo Clinic, Jacksonville, FL USA; 77 Yale University School of Medicine, New Haven, CT USA; 78 McGill University, Montreal-Jewish General Hospital, Montreal, QC Canada; 79 Sunnybrook Health Sciences, Toronto, ON Canada; 80 University of British Columbia Clinic for AD & Related Disorders, Vancouver, BC Canada; 81 Cognitive Neurology, St. Joseph’s Health Care, London, ON Canada; 82 Cleveland Clinic Lou Ruvo Center for Brain Health, Las Vegas, NV USA; 83 Northwestern University, Chicago, IL USA; 84 Premiere Research Institute (Palm Beach Neurology), West Palm Beach, FL USA; 85 Georgetown University Medical Center, Washington, DC USA; 86 Brigham and Women’s Hospital, Boston, MA USA; 87 Stanford University, Stanford, CA USA; 88 Banner Sun Health Research Institute, Sun City, AZ USA; 89 Howard University, Washington, DC USA; 90 Case Western Reserve University, Cleveland, OH USA; 91 Parkwood Hospital, London, ON Canada; 92 Banner Alzheimer’s Institute, Phoenix, AZ USA; 93 Dent Neurologic Institute, Amherst, NY USA; 94 Ohio State University, Columbus, OH USA; 95 Albany Medical College, Albany, NY USA; 96 Hartford Hospital, Olin Neuropsychiatry Research Center, Hartford, CT USA; 97 Dartmouth-Hitchcock Medical Center, Lebanon, NH USA; 98 Wake Forest University Health Sciences, Winston-Salem, NC USA; 99 Rhode Island Hospital, Providence, RI USA; 100 Butler Hospital, Providence, RI USA; 101 University of South Florida, Byrd Alzheimer’s Institute, Tampa, FL USA; 102 University of Chicago, Chicago, IL USA; 103 Eastern Virginia Medical School, Norfolk, VA USA; 104 Charter Health Research Services, Chicago, IL USA; 105 Houston Methodist Neurological Institute, Houston, TX USA; 106 Barrow Neurological Institute, Phoenix, AZ USA; 107 Nathan Kline Institute, Orangeburg, NY USA; 108 Ralph H. Johnson Veterans Administration Health Care Services, Charleston, SC USA; 109 Vanderbilt University Medical Center, Nashville, TN USA; 110 University of Texas Health, San Antonio, TX USA; 111 Rutgers University, Newark, NJ USA; 112 Gonzalez & Aswad Health Services, Miami, FL USA; 113 Medical University of South Carolina, Charleston, SC USA

**Keywords:** Biomarkers, Dementia

## Abstract

**Background:**

Despite established consensus about the individuality of brain physiology and structural changes, current neuroimaging biomarker paradigms heavily rely on population averages. Here, we define normativity not as a single reference point but as a landscape of permissible configurations arising from diverse physiological manifestations.

**Methods:**

We introduce the Nearest Neighbor Normativity (N^3^) framework, designed to parse the natural heterogeneity in brain structural measurements into a diversity-aware biomarker of brain structural decline. Through repeated comparisons within different demographic subgroups, N^3^ contextualizes an individual’s measurements from several viewpoints along the aging continuum. Relying on local density estimation, it accommodates multiple, shared normative configurations within and across age groups. We use T1-weighted MRI data from 29,883 individuals for training and 7,013 individuals for validation, and benchmark our framework against Brain Age models and Normative Modeling. We evaluate each biomarker’s ability to detect neurodegenerative diseases, using them as representative models of atypical brain structural decline.

**Results:**

In group-level analyses, the N^3^ biomarker shows the largest effect sizes in the detection of Mild Cognitive Impairment, Alzheimer’s Disease, and Frontotemporal Dementia. Similarly, the N^3^ biomarker achieves the highest predictive performance in the classification of neurodegenerative diseases on a single subject level. Notably, the N^3^ estimates remain stable across age and robust to compositional changes in the reference sample. Finally, correlation analyses indicate that it captures distinct and complementary aspects of brain structural variation compared to existing approaches.

**Conclusions:**

Our findings underscore that rethinking normativity assessments to explicitly accommodate natural inter-individual variability can advance our collective efforts toward truly personalized patient care.

## Plain language summary

Brain aging varies greatly between individuals, yet standard medical tools rely on population averages that may miss important individual differences. We developed a method called Nearest Neighbor Normativity (N^3^) that assesses brain health by searching for similar configurations along the aging spectrum and their likelihood, rather than to a single average. Using MRI scans from nearly 37,000 people, we tested whether this framework could detect brain changes associated with mild cognitive impairment, Alzheimer’s disease and frontotemporal dementia. The N^3^ method outperformed existing approaches, distinguishing disease-related brain changes from natural aging effects more accurately. This work suggests that personalized medical assessments accounting for natural human diversity may improve our ability to detect brain diseases early and support more targeted interventions.

## Abstract (web summary)

Leenings et al. methodologically reformulate neuroimaging standards to explicitly account for natural inter-individual variability of brain physiology. Applied to brain structural decline, the result is a diversity-aware biomarker which improves the detection of disease signals in both statistical and machine learning analyses.

## Introduction

Finding (subtle) individual norm deviations moderates our ability to diagnose and treat diseases effectively and is a practical necessity for the personalization of patient care. To this end, neuroimaging biomarkers hold the promise of providing objective indices of neurophysiological health^1–6^. They are closely tied to the concept of brain structural normativity, i.e., the degree of alignment with norms seen in the general population. Despite established consensus about the individuality in brain structure and aging patterns, however, current neuroimaging biomarkers heavily rely on population averages in their quantifications. They thereby exclude the possibility of multiple, equally viable normative health states, potentially obscuring tailored medical interventions to individual physiology^7,8^. Redefining neuroimaging biomarkers is thus not merely an academic exercise, but holds profound implications for medical practice.

Two prominent approaches have emerged in the search for reliable neuroimaging biomarkers in brain structure thus far. The first approach, called normative modeling, uses statistical distributions to quantify brain structural measures in relation to the population average and the variance around it^9,10^. It has been successfully applied to detect brain structural norm deviations in various psychiatric disorders^11^. By design, normative models interpolate natural variability into a single reference distribution, centered around the population average. This mean-centric framework neglects the heterogeneity of physiological manifestations and does not account for biologically valid alternative norms. Thus, these population-wide, and often univariate, models risk masking subtle details that are critical for obtaining nuanced individual clinical insights^12^.

The second approach, called Brain Age^13,14^, is a derivative of normative modeling and trains machine learning models to predict chronological age from brain structure using examples of a healthy reference sample. The resulting biomarker, the Brain Age Gap (BAG), is defined as the difference between chronological and predicted age and has been statistically associated with numerous neurological and psychiatric conditions, such as Alzheimer’s Disease (+5-10 years), Mild Cognitive Impairment, (+1-10 years), Major Depressive Disorder (BAG 1-4 years), Schizophrenia (+3-12 years)^15–19^.

However, the BAG’s ability to capture individual norm deviations is naturally limited due to the multifaceted nature of individual aging processes. How quickly aging effects progress in individuals is highly variable and a complex interplay of genetic predisposition, behavioral choices, and the cumulative impact of various adverse or protective exposures^20–24^. This unique combination of factors inevitably results in different aging effects seen among same-aged individuals. Using chronological age to assess normative aging effects thus inherently lacks precision. Consequently, the resolution of the BAG as a personalized brain structural assessment tool is limited, and its utility is mainly constrained to group comparisons.

**Fig. 1:**
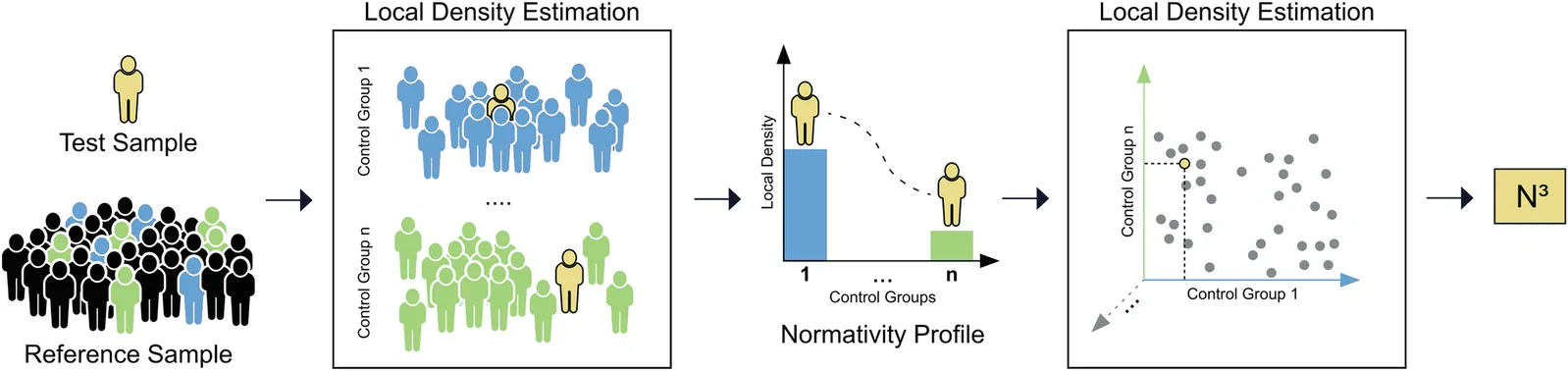
Schematic summary of the nearest neighbor normativity N^3^ framework. The N^3^ framework analyzes the local sample density around individual measurements in strategically chosen subgroups of a normative reference dataset. This strategy accommodates multiple, equally viable health states in the population and promotes the detection of nuanced irregularities that may escape broader comparative models. Moreover, by evaluating the local sample density in multiple strategically chosen subgroups, the N^3^ framework provides a multifaceted contextualization of medical observations. We call the resulting vector a normativity profile. The information contained in the normativity profile is converted into a singular, actionable metric by re-applying local density estimation. The resulting metric expresses how common a normativity profile is for a particular demographic or clinical subgroup. It can be adapted to a variety of clinical inquiries, such as how common this profile is amongst young women, treatment-resistant patients, or genetic predispositions.

To address the methodological shortcomings of the approaches described above, we propose a normativity estimation framework called Nearest Neighbor Normativity (N^3^) (see Fig. 1).

Normativity in our framework is defined as a structured space of permissible configurations that arises from the diverse physiological manifestations observed in healthy individuals. Healthy inter-individual variability reflects biologically principled movement in this space, whereas anomalies correspond to configurations that deviate in biologically unexpected directions. A biomarker should be able to distinguish physiologically coherent variants from truly atypical patterns that depart from the manifold of healthy configurations. Grounded within this conceptualization, the N^3^ framework shifts from estimating distance to an average, prototypical observation toward locating each individual within a high-dimensional landscape of plausible and implausible configurations.

In this work, we introduce the N^3^ framework and use it to assess the normativity of brain structures while explicitly accounting for the natural and systematic changes along the aging continuum. By relying on context-enriched assessments, the N^3^ framework is designed to improve the understanding of variance between individual brain structures within the natural diversity seen along the aging continuum. Specifically, the N^3^ framework employs local density estimation within demographic subgroups, which allows multiple plausible biological configurations to co-exist within the context of an age- and sex-matched population. The local sample density estimation captures natural heterogeneity while the anchoring to demographic subgroups ensures that comparisons occur among biologically similar individuals and remain clinically interpretable. We use a set of global volumetric biomarkers (gray matter, white matter, white matter hyperintensities, cerebrospinal fluid, and total intracranial volume) to demonstrate conceptual feasibility and benchmark the resulting N^3^ biomarker relative to conventional neuroimaging biomarker approaches, including conventional normative modeling and Brain Age models. As a representative use case for benchmarking, we focus on neurodegenerative disorders, which are characterized by structural alterations and deviations from normative aging trajectories, and therefore provide a suitable testbed for the evaluation of the proposed framework.

## Methods

### Materials

#### Training data

T1-weighted MRI scan data from six different studies were provided by the respective consortia. The training data for fitting models for the different biomarkers comprised 30,047 samples from the NAKO cohort^25–27^. The German National Cohort (NAKO) is a population-based longitudinal study initiated in 2014 aiming to investigate the risk factors for major chronic diseases in 200,000 persons living in Germany. It contains neuroimaging data from participants spanning a broad age range. We exclude age groups below 22 years and above 72 years due to small sample sizes (n  < 100), which restricts the final sample to 29,883. In this study, we utilize the participants’ 3.0-Tesla T1w-MPRAGE MRI scans (voxel size 1 × 1 × 1 mm3, repetition time/ echo time=2300/2.98, flip angle=9^∘^)^25–27^. The NAKO study protocol was approved by ethics committees of participating German universities and medical centers and a leading ethics vote of Bayerische Landesärztekammer (13023 on 27 March 2013). Access to the NAKO data was authorized for R. Leenings by the responsible principal investigators.

#### Testing data

The testing data comprised 7013 additional T1-weighted MRI scans from independent study populations. We validate each biomarker’s ability to differentiate between healthy inter-individual variability and pathological norm deviations. To this end, instances of Mild Cognitive Impairment (MCI), Alzheimer’s Disease (AD), and Frontotemporal Dementia (FTD) serve as model diseases to represent brain structural alterations and pathological norm deviations. Data were sourced from the Alzheimer’s Disease Neuroimaging Initiative (ADNI)^28^, the Australian Imaging, Biomarker & Lifestyle Study of Aging (AIBL)^29^, the Frontotemporal Lobar Degeneration Neuroimaging Initiative (NIFD), and the Open Access Series of Imaging Studies 3 (OASIS3)^30,31^ (for details see Table 1). All datasets were collected with ethical approval and informed consent from participants. In general, if more than one measurement was available per participant, we restricted each study’s dataset to the first (baseline) measurement of the participant. Exclusion criteria were applied based on age; participants younger than 22 years or older than 72 were omitted from the study due to insufficient sample sizes in the normative reference sample.

**Table 1:** Study Data Summary

Study	Group	N	Mean Age	Sex
ADNI	HC	1073	68.36 ± 3.3	634 females (59.09%)
	MCI	1529	66.71 ± 4.25	729 females (47.67%)
	AD	588	67.2 ± 4.65	291 females (49.48%)
AIBL	HC	368	68.00 ± 2.77	217 females (58.97%)
	MCI	78	68.05 ± 3.54	33 females (42.31%)
	AD	28	66.89 ± 4.44	16 females (57.14%)
OASIS3	HC	1643	63.36 ± 6.85	1028 females (62.57%)
	MCI	63	66.67 ± 4.85	37 females (58.73%)
	AD	228	66.54 ± 4.94	97 females (42.54%)
NIFD	HC	263	62.71 ± 6.41	148 females (56.27%)
	FTD	317	63.26 ± 5.66	120 females (37.85%)
MACS	HC	835	35.71 ± 12.6	528 females (63.23%)
NAKO	HC	29883	48.45 ± 12.09	13201 females (44.18%)

T1-weighted MRI scans from six different studies were provided by the respective consortia. If more than one measurement was available per participant, we restrict each study’s dataset to the first (baseline) measurement of the participant. Exclusion criteria were applied based on age; participants younger than 22 years or older than 72 were omitted from the study due to insufficient sample sizes in the normative reference sample (NAKO).

##### Alzheimer’s disease neuroimaging initiative (ADNI)

ADNI is a major multicenter study started in 2003, designed to develop clinical, imaging, genetic, and biochemical biomarkers for the early detection and tracking of Alzheimer’s disease. The ADNI was launched as a public-private partnership, led by Principal Investigator Michael W. Weiner, MD. The primary goal of ADNI has been to test whether serial MRI, positron emission tomography (PET), other biological markers, and clinical and neuropsychological assessment can be combined to measure the progression of neurodegeneration. The original ADNI protocol and participant-information documents were approved by the institutional review board or equivalent research ethics committee at each participating recruitment institution before study initiation. The participating institutions are listed in the official ADNI acknowledgement list. All participants or their authorized representatives provided written informed consent. R. Leenings was granted access to the de-identified data in accordance with the ADNI data-use agreement and access requirements. We included 1.5 and 3.0-Tesla T1w-MPRAGE MRI scans adhering to the ADNI sequence protocol; for scanner-specific details, please see https://adni.loni.usc.edu/data-samples/adni-data/neuroimaging/mri/mri-scanner-protocols/).

##### Australian imaging, biomarker & lifestyle study of aging (AIBL)

AIBL is an Australian study launched in 2006 focusing on understanding the pathways to Alzheimer’s disease. The cohort includes participants diagnosed with Alzheimer’s disease, mild cognitive impairment, and cognitively unimpaired elderly participants, providing insights into the aging process and the development of neurodegenerative diseases. AIBL study methodology has been reported previously^32^. AIBL was approved by Austin Health Human Research Ethics Committee (HREC), and all participants provided written informed consent. R. Leenings was granted access to the de-identified data in accordance with the AIBL data-use agreement and access requirements. MRI scans were performed using a 3D MPRAGE image (voxel size 1.2 × 1 × 1 mm3, repetition time/echo time=2300/ 2.98, flip angle=8^∘^)^29^.

##### NIFD dataset

The Frontotemporal Lobar Degeneration Neuroimaging Initiative (FTLDNI) was funded through the National Institute of Aging and started in 2010. The primary goals of FTLDNI were to identify neuroimaging modalities and methods of analysis for tracking frontotemporal lobar degeneration (FTLD) and to assess the value of imaging versus other biomarkers in diagnostic roles. We use the provided 3D MPRAGE T1-weighted images (voxel size 1 × 1 × 1 mm3, repetition time/echo time=2300/2.9, matrix = 240  × 256  × 160). The Principal Investigator of NIFD was Dr. Howard Rosen, MD, at the University of California, San Francisco. The NIFD/FTLDNI study protocol was approved by the relevant institutional review boards at participating sites, including the University of California, San Francisco, University of California, Berkeley, Lawrence Berkeley National Laboratory, and Mayo Clinic, Rochester. Written informed consent was obtained from all participants or their legally authorized representatives. R. Leenings was granted access to the de-identified data in accordance with the NIFD data-use agreement and access requirements. The data are the result of collaborative efforts at three sites in North America. For up-to-date information on participation and protocol, please visit http://memory.ucsf.edu/research/studies/nifd.

##### Open Access Series of Imaging Studies 3 (OASIS3)

OASIS3 serves as a comprehensive digital repository for MRI brain data that supports longitudinal studies of normal aging and cognitive decline^30,31^. The project is distinguished by its wide age range of participants, providing diverse datasets that enhance the understanding of late-life brain diseases alongside physiological aging processes. We include 3D MPRAGE T1-weighted images (voxel size 1.0 or 1.2 × 1 × 1 mm3, repetition time/echo time = 2300/2.95 or 2400/3.16 (depending on the scanner), flip angle = 9^∘^, FoV = 240 or 256 mm). OASIS-3 was approved by the Washington University School of Medicine IRB. All participants provided written informed consent. R. Leenings was granted access to the de-identified data in accordance with the OASIS-3 data-use agreement and access requirements.

##### Marburg-Münster affective disorder cohort study (MACS)

The MACS cohort is part of the DFG-funded research group FOR2107 cohort, researching the etiology and progression of affective disorders^33^. The goal is to integrate and understand the clinical and neurobiological effects of genetic and environmental factors, and their complex interactions. We use the T1-weighted neuroimaging scans of n=835 healthy control participants to evaluate the stability of the N^3^ models. Images were acquired in Marburg (MR) or Münster (MS) (voxel size 1 × 1 × 1 mm3, repetition time/echo time=MR: 1900, MS: 2130/MR: 2.26, MS: 2.28, flip angle=8^∘^, FoV = 256 mm, matrix = 256  × 256, slice thickness = 1 mm). MACS was approved by ethics committees at the University of Münster and Marburg, with informed consent obtained from all participants. Access was authorized for R. Leenings by the responsible principal investigators. Participants received financial compensation.

#### Image preprocessing

All images underwent preprocessing using the standard software CAT12 (version: cjp_v0008, spm12 build v7771; cat12 build r1720) with default parameters. In short, images were bias-corrected, tissue classified, and normalized to MNI-space using linear and non-linear transformations. In this work, we use volume metrics of gray matter (GM), white matter (WM), white matter hyperintensities (WMH), total intracranial volume (TIV), and cerebrospinal fluid (CSF) derived from the SPM12-based tissue segmentation procedure.

### N^3^ algorithm

The N^3^ framework evaluates medical measurements within intentionally selected subgroups of a reference dataset to realize comparisons from multiple, clinically meaningful perspectives (see Table 2). Within these subgroups, N^3^ quantifies normativity via local sample density estimation, determining how frequently similar value combinations occur within local neighborhoods of a measurement space. Multiple density peaks can exist within a single neighborhood, so that, for example, an age and sex group may contain several plausible normative patterns. Rather than averaging across these configurations, N^3^ retains their plurality, allowing each individual’s configuration to be compared against the most appropriate local reference.

**Table 2:** Notation and variable definitions

Symbol	Definition
$X\in {{\mathbb{R}}}^{a\times b}$	Reference dataset with *a* samples and *b* features
${X}_{c}\in {{\mathbb{R}}}^{n\times m}$	Subgroup *c* with *n* samples and *m* features, *c* ∈ {1, …, *g*}
*q*	Query sample being evaluated
*λ*(*q*, *c*)	Local sample density: inverse of average distance to *k* nearest neighbors for sample *q* in subgroup *c*
Λ_*c*_	Set of all local densities in subgroup *c*
${{\Lambda }}{\prime}$	Median-normalized densities across all subgroups
*ℓ*(*q*, *c*)	Likelihood of normalized density
ℓ^*^(*q*, *c*)	Signed likelihood, scaled to [ − 1, 1]
*ϕ*(*q*)	Normativity profile: vector of signed likelihoods across subgroups

#### Local density estimation in subgroups

The algorithm quantifies the local sample density in different subgroups, defined as submatrices ${X}_{c}\in {{\mathbb{R}}}^{n\times m},c=1,2,\ldots ,g$ of a reference dataset $X\in {{\mathbb{R}}}^{a\times b}$. We normalize each submatrix *X*_*c*_column-wise using min-max scaling so that all entries satisfy *X*_*cij*_ ∈ [0, 1] for *i* = 1, 2, …, *n*, *j* = 1, 2, …, *m*.

Within each subgroup, we use a Nearest Neighbor approach and define a subset *N* for each sample *q*, such that it contains the *k* points in *X*_*c*_ that are the closest to sample *q*^34,35^. Distance *d* is measured using the Euclidean distance. We define the local sample density *λ*(*q*, *c*) of sample *q* in subgroup *c* as the inverse of the average distance to its *k* nearest neighbors.

1 $$\lambda ({q}_{i},c)={\left(\dfrac{1}{k}{\sum }_{j = 1}^{k}\parallel {x}_{j}-q\parallel \right)}^{-1}$$

We calculate the local sample densities *λ* as described above for all samples in a subgroup *c* and get Λ_*c*_ = {*λ*(*q*_*i*_, *c*)∣*i* = 1, 2, …, *n*}.

To ensure comparability between the different subgroups, we divide Λ_*c*_ by the median value.

2 $${{{\Lambda }}}_{c}=\dfrac{{{{\Lambda }}}_{c}}{{{\rm{median}}}\left({{{\Lambda }}}_{c}\right)}$$

As a result, we have a set of normalized local sample density estimations for *g* subgroups ${{\Lambda }}{\prime} =\{{{{\Lambda }}}_{c}| c=1,2,\ldots ,g\}$.

To establish global context across the *g* subgroups, we aggregate the normalized local density estimates and model their empirical distribution using a parametric framework. Specifically, we fit the exponentiated Weibull distribution^36^ to the values in ${{\Lambda }}{\prime}$, leveraging its flexibility to accommodate diverse distributional morphologies including skewness, heavy tails, and multimodality commonly observed in density-based statistics.

The probability density function of the exponentiated Weibull distribution is given by:

3 $$f(x;\alpha ,\beta ,\gamma )=\dfrac{\gamma \alpha }{\beta }{\left(\dfrac{x}{\beta }\right)}^{\alpha -1}\exp \left[-{\left(\dfrac{x}{\beta }\right)}^{\alpha }\right]{\left\{1-\exp \left[-{\left(\dfrac{x}{\beta }\right)}^{\alpha }\right]\right\}}^{\gamma -1},$$

where $x\in {{\mathbb{R}}}^{+}$ denotes the normalized local density estimate, *α* > 0 is the shape parameter governing distributional skewness, *β* > 0 is the scale parameter, and *γ* > 0 is the exponentiation parameter controlling tail behavior. We denote the complete parameter vector as *θ* = (*α*, *β*, *γ*)^⊤^ and estimate it via maximum likelihood estimation. Using the fitted distribution *f*( ⋅ ; θ^*^), we transform each normalized density estimate into a likelihood value, thereby establishing a common probabilistic scale across all subgroups and samples.

4 $$\ell (q,c)\ =\ f\,(\lambda {\prime} (q,c)\,;\,{\theta }^{* })$$

where *f*( ⋅ ; *θ*) denotes the exponentiated Weibull density with parameters *θ*, θ^*^ are the fitted parameters, and *ℓ*(*q*, *c*) is the likelihood assigned to the normalized local density of sample *q* in subgroup *c*.

To keep as much information as possible, we add a sign to *f*, which indicates in which direction a sample is deviating from the median. In this context, samples whose local sample density is smaller than the median receive a negative value, while samples whose local sample density is larger than the median have a positive value.

5 $${\ell }^{* }(q,c)=\left\{\begin{array}{l}-\ell (q,c)\quad \,{\mbox{if}}\,\,\lambda (q,c) < {{{\rm{median}}}}_{c}\quad \\ \ell (q,c)\quad \,{\mbox{otherwise}}\hfill\,\quad \end{array}\right.$$

Finally, to foster intuitive interpretation, we scale the signed likelihood *ℓ* to an interval of [-1, 1]. The final value is a normativity estimation on how common the configuration of sample *q* appears within a particular control group *c*.

#### Normativity profile and N^3^ biomarker

In the next step, we aggregate normativity estimations from several subgroups into a vector, which we call normativity profile and which depicts an individual’s normativity measured from multiple meaningful angles or viewpoints.

6 $$\phi (q)=\{{\ell }^{* }(q,{c}_{1}),{\ell }^{* }(q,{c}_{2}),\ldots ,{\ell }^{* }(q,{c}_{g})\}$$

To synthesize the multidimensional information entailed in an individual normativity profile into a single, actionable metric, we repeat the local density estimation approach outlined above. In a second layer of normativity estimation, we use the normativity profile *ϕ* as input data and repeat the local sample density estimation approach outlined above. Now, the local density estimation is based on the *g* values of *ϕ*, computed in the normativity profile space. Thereby, we measure the commonness of a normativity profile in relation to other normativity profiles seen in a particular reference population. This can either be done globally (on all normativity profiles of the sample), or again in subgroups. The output of this meta-normativity estimation is the return value of the N^3^ algorithm, what we call the N^3^ biomarker.

#### Training vs. inference phase

The N^3^ algorithm is trained using a normative reference sample. Using this sample, we learn scaling parameters, neighborhood structure, and distributional parameters, first in the original measurement space and second in the normativity profile space. Specifically, we first use the values in the reference sample to learn how frequently different medical configurations occur within each subgroup. We then aggregate the resulting values into normativity profiles and fit a second layer of local density estimation on these profiles.

At inference, a new individual is projected into the learned spaces. We apply the subgroup definitions, the scaling parameters estimated during training, and compute the local densities and signed likelihoods using the pre-fitted distributions. During this process, we first obtain a normativity profile, which is subsequently passed through the second-layer models, again using only parameters learned in the training phase, to yield the individual’s N^3^ biomarker value.

#### Application to brain structure

In our application to brain structure, the local density estimation is applied across five volumetric summary measures (gray matter, white matter, white matter hyperintensities, cerebrospinal fluid, total intracranial volume) to identify normative configurations. While this dimensional reduction enables a robust prototyping of the framework and facilitates validation across large heterogeneous cohorts, the framework is not inherently limited to these measures, and extensions to whole-brain representations are considered in the Discussion.

We stratify the training sample by sex and age, resulting in a total of 100 demographic groups containing same-aged females or males (22 to 72 years), respectively. To mitigate unequal sample sizes across age groups (due to cohort age distribution), we implemented a joining procedure where underrepresented age groups (defined as having *n* < median sample size per sex) were merged with the adjacent 1-year age band. The merging procedure first joined the immediately lower neighboring age group, then, if still underrepresented, joined the upper neighboring group. As adjacent age groups share similar brain structural characteristics, this strategy supports smooth normative transitions across the aging spectrum and ensures comparable variability in the neighborhoods across the aging continuum.

Within each demographic neighborhood, the neighborhood in the five-dimensional biomarker space was characterized by local sample density computed using k-nearest neighbors, where $k=\min (\,{\mbox{round}}(0.1\times {n}_{{{\rm{neighborhood}}}}),15)$. The 10% rule ensures that k scales with neighborhood size while the upper bound of *k* = 15 prevents excessively broad comparisons that would override fine-grained local density structure.

The N^3^ algorithm proceeds in two steps. First, for each individual, we compute local sample density estimates within each age-year neighborhood across the entire continuum (ages 22-72), generating 50 local density values (one per year per sex). These are combined into a *normativity profile* that captures the individual’s normativity across the entire aging spectrum. Balancing the variability in the neighborhoods across the aging continuum is relevant to this first level of local sample density estimation, as all estimates are then jointly evaluated in the resulting normativity profile. In contrast, in the second layer of local density estimation, the individual’s normativity profile is evaluated within the same-aged and same-sex peer group only, deriving the final N^3^ biomarker. This two-step structure ensures that individuals are first contextualized within the broader aging continuum, then assessed relative to exact age- and sex-matched peers.

### Brain age model

In the Brain Age paradigm, the brain structure is evaluated with respect to aging effects seen in a healthy reference sample. This is realized by means of a machine learning model trained to predict chronological age from brain structure. The deviation between chronological and predicted age is referred to as the Brain Age Gap (BAG). While a small BAG is considered normative and age-appropriate, a larger positive or negative BAG symbolizes premature or delayed neurostructural degeneration, respectively. The resulting normativity estimation, i.e., the BAG values, has been associated with numerous neurological and psychiatric conditions^15,16^. For comparison with N^3^, we train a Brain Age Model using the Python library photonai^37^. We use a Support Vector Machine (SVM), for which we optimize the C and gamma parameters in the nested cross-validation procedure (k=10 outer folds and two randomly shuffled inner folds with a test size of 0.1). The best model achieves an average MAE of 5.43. We use age-stratified 90% of the available normative dataset for model training. We use the remaining 10% of the normative dataset to train a linear age bias correction as described in Peng et al.^38^.. For the evaluation of unseen samples, we use the Brain Age SVM model to predict age and subsequently apply the bias correction model before we calculate the difference between the chronological and predicted age, the BAG.

### Normative modeling

We calculate normative models on the training data using the Predictive Clinical Neuroscience toolkit as described in Rutherford et al.^10^.. To train the models, we normalize GM, WM, WMH, and CSF by Total Intracranial Volume (TIV) and fit Bayesian Linear Regression models with default parameters. Subsequently, we derive z-scores for each variable and aggregate them into four normative modeling markers, each yielding a single score per individual. The best-performing marker counts the number of absolute z-scores greater than 1.96, reflecting the burden of outlying features per person, and is discussed throughout the manuscript. We evaluated three more aggregation strategies. The second marker sums the absolute z-scores across all features, capturing the overall magnitude of deviation from the normative reference. The third marker is defined as the average z-score, summarizing the mean direction and extent of deviation. Finally, we obtain a low-dimensional embedding of the multivariate z-score patterns using a UMAP component, which serves as a fourth, non-linear aggregation of normative deviations. For completeness, Supplementary Tables S3 and S4 report the statistical and machine-learning performance of all evaluated markers.

### Statistical analysis

A Type III Sum of Squares ANOVA was performed using an ordinary least squares model to assess the discriminative and explanatory power of each normativity marker in distinguishing patients from controls. The model was adjusted for potential confounders, including age, age squared (to mitigate non-linear effects), sex, and scanner. Partial eta squared (*η*^2^) was used to quantify effect size, providing an estimate of how much variance in disease progression could be explained by each normativity marker. Post-hoc pairwise comparisons of effect sizes were conducted using two-tailed permutation testing (1,000 random permutations). *P*-values were corrected for multiple comparisons using the Benjamini-Yekutieli false discovery rate method, controlling for dependency across comparisons.

To assess each normativity marker’s consistency across age groups, an analysis of age bias was conducted using Spearman’s rank correlation to evaluate the correlation between the normativity estimation values and age in healthy controls.

To assess stability of the N^3^ models, the Intraclass Correlation Coefficient (ICC) model (2,1) was computed for models trained on artificially downsampled subgroups of the NAKO study. Particularly, we divide the training set in k=[10, 5, 3, 2] non-overlapping parts of equal size, train normativity models within each of these subsets, and use validation data to ensure the stability of the normativity estimates. Validation subsets included n=835 healthy control participants from the Münster-Marburg Affective Disorder Cohort (MACS)^33^ study, which predominantly comprises younger and middle-aged adults, and an additional n=1073 healthy older adults from the ADNI study to span a wider age demographic. To ensure the validity of the test, we used only age groups with more than 500 samples available from the training sample and more than 20 samples in the test sample.

All statistical analyses were implemented in Python using the *scipy*, *statsmodels* and pingouin libraries.

### Machine learning analysis

Machine learning models transcend conventional statistical models by handling multivariate and non-linear relationships and shifting the focus from group average comparisons to predictions on an individual level^39^. We employ cross-validation strategies, which systematically test each marker against new, unseen data to verify the accuracy, robustness, and generalizability of the models^40^. Our analytical pipeline employed the open-source Python framework photonai^37^. The analysis involved nested cross-validation to robustly estimate model performance and avoid overfitting, using k=5 outer folds and k=10 inner folds, each fold stratified to entail a balanced proportion of samples from the diseased class. Hyperparameter optimization was performed via Grid Search to fine-tune the support vector machine (SVM) parameters C and gamma. The machine learning pipeline included steps for z-normalization and balanced sampling (random under-sampling techniques) to address class imbalance within the training data. Models were trained with each of the biomarkers as the only feature, enabling us to quantify each biomarker’s ability to capture disease signals of neurodegenerative diseases. We measure balanced accuracy, recall, precision, and f1 score of each of the normality markers in the classification of neurodegenerative diseases.

## Results

### The N^3^ biomarker for brain structure and its biological interpretation

**Fig. 2:**
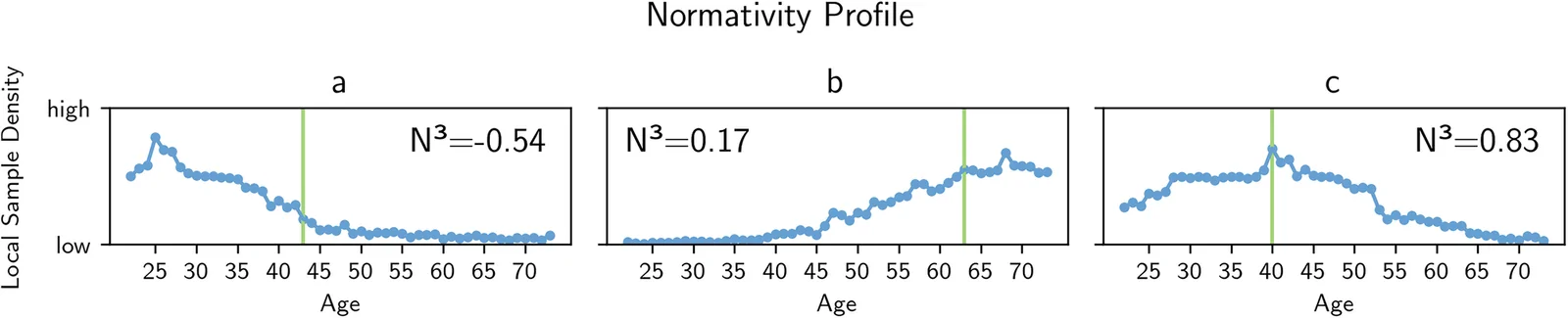
Exemplary normativity profiles of three individuals. For illustration, we show the normativity profiles of three individuals of the training sample. The N^3^ framework compares an individual’s brain structural features (global volumes of gray matter, white matter, white matter hyperintensities, cerebrospinal fluid and total intracranial volume) to those seen in different age groups along the age continuum. The normativity profile shows the local sample density in different age groups, i.e., how common similar volumetric configurations appear along the aging continuum (blue) as well as the individual’s chronological age (green). **a** An individual’s data show higher local sample density in younger age groups, indicating that the volumetric configuration is more common within younger age groups and less common within same-aged individuals **b** An individual’s volumetric configuration appears more common within older age groups, indicating premature aging effects. **c** A brain structure appears most common within its own age group and decreasing in occurrence within other age groups.

In a first step, the N^3^ framework analyzes the frequency of different brain structural configurations within sex-matched subgroups of successive chronological age. The result is a vector that expresses how commonly an individual’s specific configuration occurs within each demographic subgroup, i.e., how their measurements are embedded within the natural variety seen along the aging continuum. We call this vector a normativity profile. Its shape indicates how an individual’s configuration is positioned relative to expected trajectories of brain structural decline. For example, an individual’s configuration may show similarity with those occurring frequently in younger age groups, indicating younger brain features and fewer aging effects as commonly seen in same-aged individuals (see Fig. 2). However, normativity profiles that shift away from an individual’s chronological age are not necessarily indicative of disease. Since genetic and environmental influences on brain structure accumulate over time, we expect substantial variability in normativity profile shapes, particularly in older age groups. We therefore quantify how frequently different normativity profile shapes occur within each age group, thereby allowing for several normative variants of brain structural decline. In a second step, the N^3^ framework therefore assesses how common a given profile shape is within each sex- and age-specific subgroup. The resulting metric is the N^3^ biomarker, which provides an individualized index of how typical or atypical a person’s brain structural configuration appears relative to the variability observed in both neurophysiology and aging trajectories across individuals.

### Increased statistical explanatory power in distinguishing neurodegenerative diseases

**Fig. 3:**
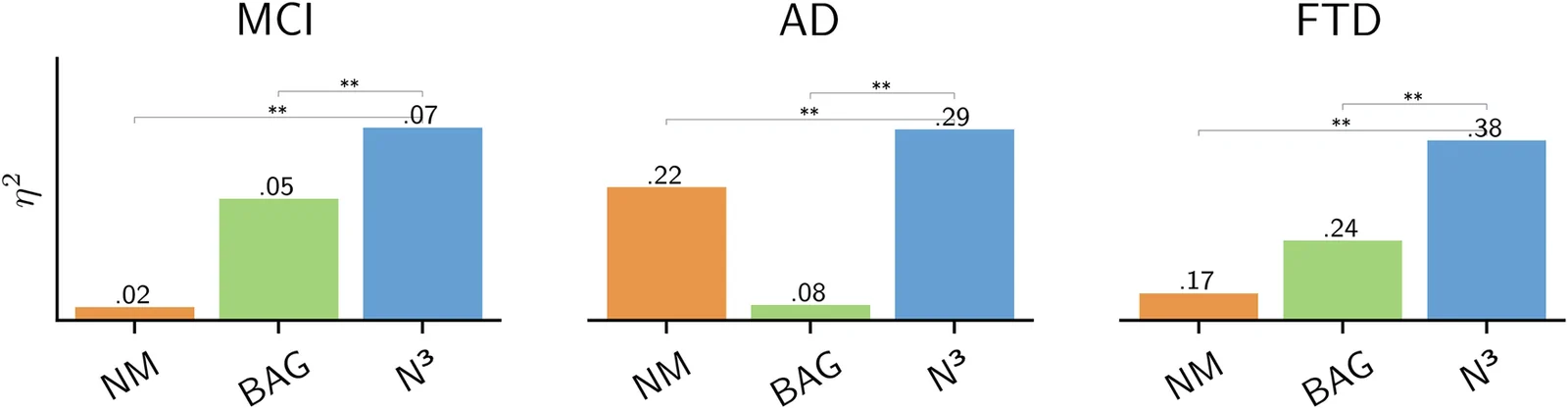
Results of the statistical analyses. Statistical effect sizes (partial eta squared - *η*^2^) are given for the different biomarkers (N^3^ - our approach, NM - Normative Modeling outlier score, and the BAG - Brain Age Gap). We evaluate each approach’s ability to differentiate between controls and diseased individuals using neurodegenerative diseases as representative model diseases, in particular Mild Cognitive Impairment (MCI), Alzheimer’s Disease (AD), and Frontotemporal Dementia (FTD) using a Type III ANOVA. Two-tailed post-hoc permutation tests revealed significantly larger explained variance of our N^3^ marker in all neurodegenerative conditions (Benjamini-Yekutieli FDR-corrected, all *p* < . 001).

For AD, the N^3^ marker showed the largest effect size (*η*^2^= 0.29), indicating that approximately 29% of the variability between the AD group and controls can be explained by differences in the N^3^ scores (see Fig. 3 and Table 3). In the context of FTD, all markers demonstrated large effect sizes, while the N^3^ stood out with an effect size of *η*^2^ = 0.38. The results for MCI differed, as all markers showed generally low explanatory power. The N^3^ marker displayed a relative advantage, with an effect size of *η*^2^ = 0.07, compared to *η*^2^ = 0.05 for the BAG and *η*^2^ = 0.02 for the normative modeling score. Overall, the N^3^ biomarker showed the largest effect sizes across all examined neurodegenerative conditions.

**Table 3:** Statistical discrimination of neurodegenerative diseases

	MCI		AD		FTD	
Marker	F	*η*^2^	F	*η*^2^	F	*η*^2^
NM	F(1,4565)=74	0.016	F(1,3709)=1073	0.223	F(1580)=121	0.173
BAG	F(1,4565)=220	0.046	F(1,3709)=328	0.081	F(1,580)=184	0.242
N^3^	F(1,4565)=**313**	**0.067**	F(1,3709)=**1412**	**0.292**	F(1,580)=**346**	**0.376**

We evaluate the markers’ ability to distinguish inter-individual variability from pathological norm deviations in statistical analyses. We evaluate each approach’s ability to differentiate between controls and diseased individuals using neurodegenerative diseases as representative models diseases, in particular *MCI* Mild Cognitive Impairment, *AD* Alzheimer’s Disease, and *FTD* Frontotemporal Dementia. We compare N^3^ - our approach, *NM* Normative Modeling outlier score, and the *BAG* Brain Age Gap, respectively. All F-statistics and effect sizes *η*^2^ are significant (*p* < 0.001). Best performance is indicated in bold.

### Single-subject predictions

**Fig. 4:**
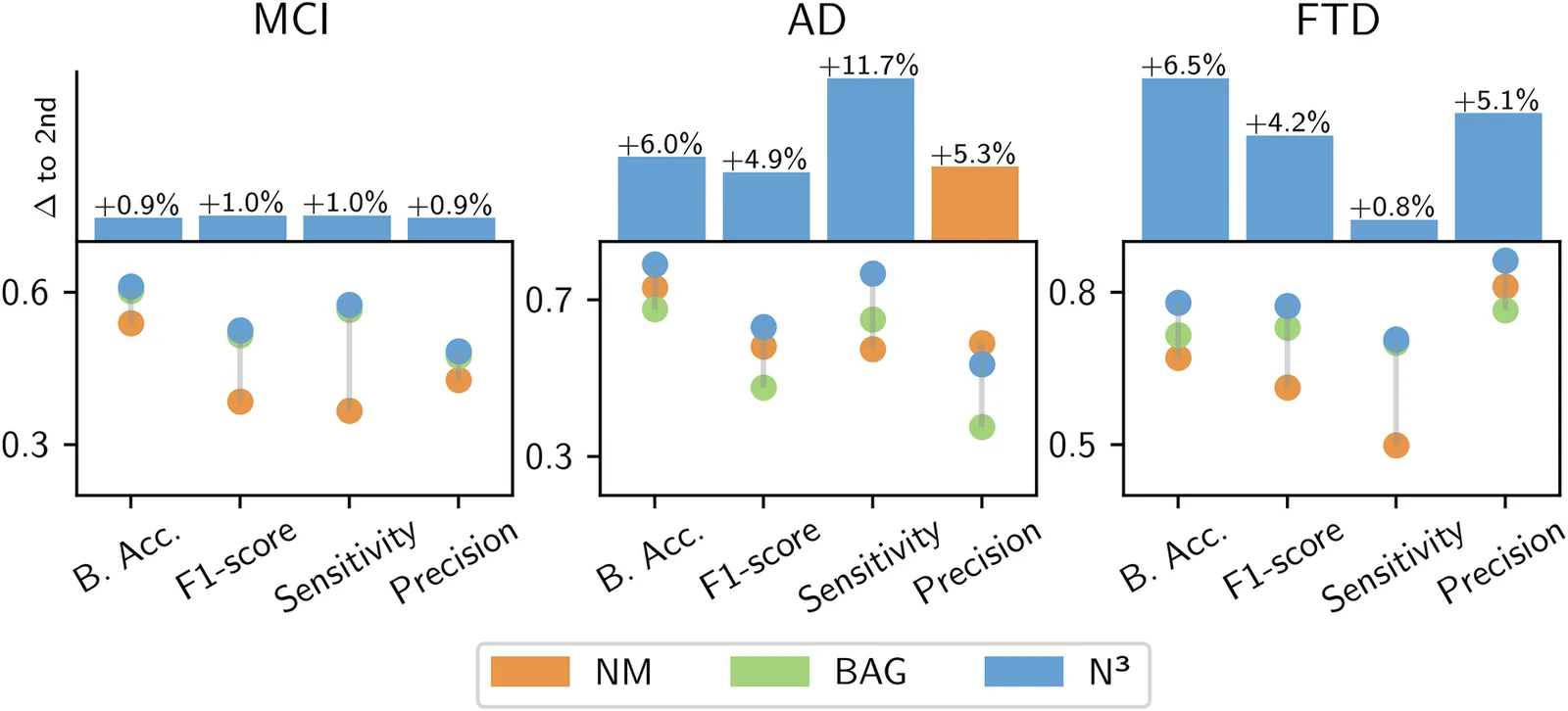
Results of machine learning analyses. We use machine learning to evaluate the expressiveness of each biomarker on a single-subject level (N^3^ - our approach, NM - Normative Modeling outlier score, and the BAG - Brain Age Gap). We evaluate each approach’s ability to differentiate between cognitively unimpaired and diseased individuals using neurodegenerative diseases as representative disease models, in particular Mild Cognitive Impairment (MCI), Alzheimer’s Disease (AD), and Frontotemporal Dementia (FTD). The N^3^ marker demonstrated increased accuracy in predicting the occurrence of pathological norm deviations. We show the different markers’ performance metrics [balanced accuracy (B.Acc), F1-Score, Recall and Precision] and the performance advantage, i.e., the delta, of the best biomarker in relation to the second-best biomarker in percentage (*Δ* to 2nd).

The efficacy of the N^3^ marker was confirmed at the individual level (see Fig. 4 and Table 4). In the specific cases of AD and FTD, the N^3^ marker demonstrated improvements in balanced accuracy scores, surpassing the second-best markers by 6.0% for AD and 6.5% for FTD. For MCI, the efficacy of all markers notably declined, in line with the small effect sizes observed in statistical analysis. Here, the N^3^ reached a 0.9% improvement to the next best marker, the BAG. With regard to the F1-scores, the N^3^ marker achieved the highest performance in all neurodegenerative diseases. N^3^’s precision for AD was 5.3% behind the normative modeling marker, and it was superior by 5.1% for FTD and 0.9% in MCI compared to the second-best result. With regard to sensitivity, the N^3^ marker displayed the highest rates in AD (11.7%), and slightly improved rates in MCI and FTD (+1.0% and +0.8%). Overall, the N^3^ biomarker achieved the highest predictive performance in the classification of neurodegenerative diseases among the compared markers in our evaluations.

**Table 4:** Performance metrics of the machine learning analyses

Marker	B. Accuracy	F1-score	Sensitivity	Precision
**Mild Cognitive Impairment**				
NM	0.539 ± 0.01	0.385 ± 0.06	0.367 ± 0.09	0.427 ± 0.03
BAG	0.603 ± 0.01	0.516 ± 0.01	0.566 ± 0.03	0.475 ± 0.02
N^3^	**0.612** ± 0.01	**0.526** ± 0.01	**0.576** ± 0.03	**0.484** ± 0.02
**Alzheimer’s Disease**				
NM	0.732 ± 0.023	0.582 ± 0.031	0.575 ± 0.050	**0.590** ± 0.012
BAG	0.676 ± 0.023	0.477 ± 0.025	0.651 ± 0.054	0.376 ± 0.014
N^3^	**0.792** ± 0.020	**0.631** ± 0.022	**0.768** ± 0.045	0.537 ± 0.020
**Frontotemporal Dementia**				
NM	0.671 ± 0.077	0.613 ± 0.043	0.499 ± 0.063	0.812 ± 0.073
BAG	0.715 ± 0.076	0.731 ± 0.073	0.700 ± 0.073	0.765 ± 0.077
N^3^	**0.780** ± 0.061	**0.773** ± 0.064	**0.708** ± 0.085	**0.863** ± 0.078

We train a machine learning classifier with each of the biomarkers as a single feature to predict the occurrence of neurodegenerative diseases in individual cases. Highest performance is indicated in bold. We compare N^3^ - our approach, *NM* Normative Modeling outlier score, and the *BAG* Brain Age Gap, respectively. Best performance is indicated in bold.

### Stability and robustness

**Fig. 5:**
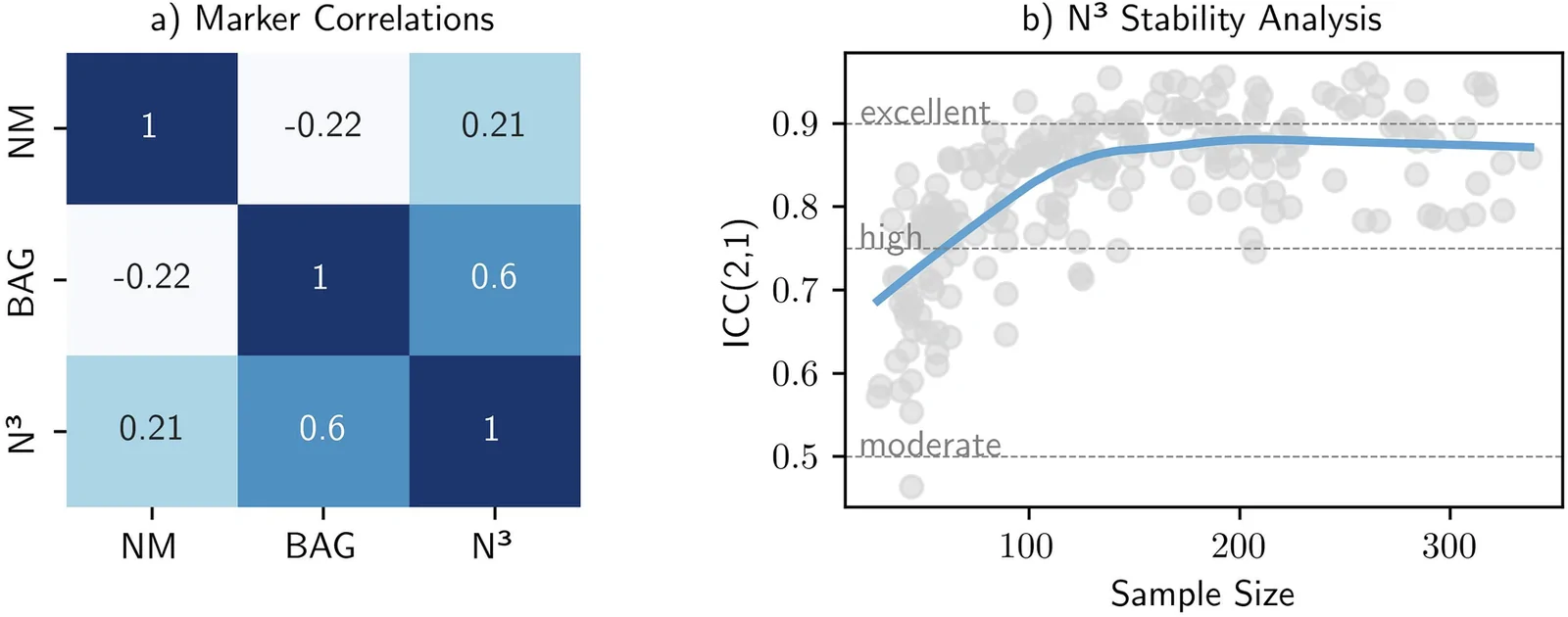
Robustness and consistency of the N^3^ framework. **a** We calculated the correlation matrix among the different biomarkers, which emphasize the distinctiveness and complementarity of the different markers. **b** We tested the impact of sample size and sample composition on the reliability of the N^3^ biomarker by calculating the intraclass correlation coefficients across repeatedly downsampled subsets of the training data. We see that the N^3^ marker exhibits an ICC≥0.75 for n > 100 individuals and approaches ICC = 0.9 for n > 150 individuals.

The calculation of the N^3^ marker relies on local density estimation. As such it is highly dependent on the composition of the reference sample. Therefore, we investigate how changes to the sample composition and sample size affect the stability of the N^3^ model. We retrained N^3^ models with downsampled subsets of varying size, thereby mimicking smaller studies and different study participants. We then apply the different normativity models and predict normativity on an external dataset. Particularly, we evaluate if predictions remain consistent across different sample sizes and sample compositions. We quantify the stability of the normativity estimates by calculating the Intraclass Correlation Coefficient (ICC) (see Methods 2.5). Results are visualized in Fig. 5b. We see that random samples of 200 individuals and above show consistently high stability (ICC of 0.75 and above). Moreover, the ICC approaches 0.9 in larger sample sizes, starting at 150 participants. While the results are calculated for the use case of brain structural normativity estimation, they are a first indication that density-estimation-based normative models can be realized by dividing larger samples into subgroups of a few hundred samples and above.

In terms of inter-marker relationships (Fig. 5a), the correlation analysis revealed generally weak associations between the normative modeling marker and the other markers (∣*ρ*∣ ≈ 0.20), indicating that these indices capture largely distinct aspects of brain structural norm deviations. By contrast, the BAG and the N^3^ marker show a moderate positive correlation (*ρ* = 0.6), suggesting that N^3^ partly aligns with established notions of accelerated brain aging while still retaining unique variance. Taken together, these patterns point to a set of complementary biomarkers with overlapping but non-redundant information content, supporting the idea that combining N^3^ with existing markers such as BAG and traditional normative indices may yield a richer, more nuanced characterization of brain structural alterations.

Finally, it is essential for normativity estimations to remain consistent across different age groups and avoid age biases that could complicate semantic interpretations. An analysis of the age correlation of the N^3^ marker indicates its stability over the age range, showing no significant association to age (*ρ*=0.009, *p* = 0.60). In comparison, the normative model marker shows a significant but smaller correlation to age (*ρ*=0.104, *p* < 0.001), while the Brain Age Gap (BAG) exhibits a moderate age bias (*ρ*=-0.21, *p* < 0.001).

Overall, these analyses show that the N^3^ biomarker yields stable normativity estimates for moderate sample sizes per age group, while remaining weakly to moderately correlated with other markers and thereby capturing partially distinct aspects of brain structural variation.

## Discussion

We have introduced the N^3^ framework, which is designed to parse the natural heterogeneity in medical observations across individuals into diversity-aware normativity assessments. We applied it to brain structure, which resulted in an informative biomarker assessing the normativity of neurophysiological measurements. The resulting N^3^ biomarker for brain structure enabled superior differentiation between natural inter-individual variability and neurodegenerative diseases.

The N^3^ framework is based on two key algorithmic principles.

First, it relies on strategic contextualization, meaning that medical measurements are compared from multiple meaningful viewpoints within intentionally chosen subgroups of a reference dataset. This promotes the detection of nuanced particularities that may escape broader comparative models and provides a multifaceted positioning of medical observations, elucidating different aspects of normativity. The resulting normativity profile can, in turn, be interrogated for a wide range of clinically relevant questions, such as how frequently a given profile occurs among young women, in treatment-resistant patients, or in individuals with specific comorbidity patterns.

Second, N^3^ assesses the normativity of medical measurements using local sample density estimation techniques. By examining how often similar value combinations occur in small neighborhoods across the measurement space, it can flexibly represent multiple co-existing normative constellations while providing a flexible and continuous measure of normativity. Based on a continuum of natural variants in physiological organization, deviations can thus be quantified with greater granularity compared to traditional approaches.

In the application of the N^3^ framework to brain structure, we chose demographic subgroups as the primary context for interpretation. In this setting, the algorithm quantifies how common a particular combination of global brain volumes is across the diversity of brain structural measurements observed among same-sex individuals along the aging continuum. The resulting vector, which we refer to as the normativity profile, encodes the resemblance of an individual’s brain to different natural atrophy progression states for that sex across the lifespan. In the second step, we contextualize each normativity profile against those of same-aged individuals to determine how frequently a given profile shape occurs within that age group. The resulting biomarker quantifies the typicality of an individual’s aging pattern among their peers. In clinical applications, the same procedure could be adapted to alternative contextualizations, such as profiles typical for specific comorbidity constellations or genetic or environmental risks.

Because N^3^ relies on local density estimation, it can represent similar aging patterns in neighboring age groups without forcing a trade-off in normativity metrics between them. More specifically, a particular configuration can be equally typical in several adjacent age groups. This contrasts with brain age models, which must assign a single best-fitting age and compress the richness of aging trajectories into a point estimate. Moreover, the N^3^ framework is able to detect irregular configurations that are uncommon across all age groups, and differentiate between general irregular anatomy and age-inappropriate configurations. Finally, the N^3^ framework is, through its conceptualization, able to reveal potential shifts towards constrained and pathologically similar configurations, if disease erodes the natural spectrum of variability and converts uniqueness into uniform manifestations.

In general, the interpretation and contextualization of individual brain structures holds significant potential for various domains. As stated above, a reliable biomarker for brain structural normativity is eagerly sought in neuropsychiatric research. Here, biomarkers may enable comprehensive assessments of neurostructural alterations associated with specific symptoms, to better understand the etiology and pathogenesis of different disease phenotypes^12,39,41^. For example, a valid and robust neurostructural biomarker would facilitate the analysis of environmental influences, genetic predisposition, or neuroinflammatory contributions to understand disease mechanics and optimize individual disease management strategies^16,23,42,43^.

In the realm of neurodegenerative diseases, the ability to detect brain structural alterations early is of critical clinical relevance, as it has been shown that structural changes in the brain can manifest well before clinical symptoms become apparent^44,45^. Furthermore, evidence supports the presence of multiple underlying neuropathological processes^21,46^, underscoring the methodological importance of models accommodating multiple disease prototypes. Here, a reliable brain structural screening tool could be attached to routine MRI scans and support disease interception to promote the prevention or delay of disease progression^47–50^.

Our approach accommodates the multivariate nature of brain structures^51^ and aligns with other modern understandings of heterogeneity, such as the concept of neurotypicality^52–54^. Traditionally seen as a uniform standard, brain architectures are now understood to encompass a spectrum of neurological function and structures, reflecting the rich diversity of human nature. Moreover, our findings resonate with recent work by Yang et al., where the authors found a range of multiple, co-occurring patterns of brain aging^55^. Their research underscores the significant inter-individual and also intra-individual variability, underscoring the complexity and uniqueness of individual neurodegenerative processes beyond population averages.

In this initial proof-of-concept study, we used a multi-site, large-scale population cohort as a reference. While it is recruited to be representative, it is a national cohort and cannot reflect the entire spectrum of biological diversity. Future work should extend this approach to multi-cohort and internationally inclusive reference datasets in order to broaden generalizability and fairness across different populations. Moreover, to advance translation to clinical practice, it is essential to evaluate how effectively normative reference models derived from study cohorts can interpret data from clinical care routines.

In general, our framework is constrained by the availability of sufficiently large reference samples to reliably estimate the frequency of different multivariate value configurations within subgroups. While large-scale population studies have become increasingly accessible, clinical investigations are often necessarily conducted in smaller, more deeply phenotyped cohorts, where resource limitations restrict sample size. In such settings, smaller studies inherently depend on the existence of a larger, demographically and clinically appropriate reference cohort to enable meaningful normative deviation estimates. In our evaluations, the N^3^ local density estimation algorithm exhibited high stability in samples of a hundred individuals, indicating substantial robustness in moderately sized research cohorts. Moreover, our framework can partially offset the limitations of smaller clinical samples by providing a principled way to interpret individual- and subgroup-level patterns relative to a broader population distribution, thereby enhancing the inferential value of these studies.

Notably, our evaluations are based on only five variables reflecting global brain structure volumes. As such, they represent the character of many clinical measurements, which are broad aggregates of more complex physiological features. In our evaluation, the N^3^ approach has demonstrated its ability to effectively decode the information contained in these limited neurophysiological variables and was able to extract more meaningful insights compared to conventional approaches. However, in future work, the N^3^ framework needs to replicate these results, for example, in the processing of high-dimensional data. To this end, deep-learning-based image representation could be used to evaluate the efficacy of the N^3^ framework in parsing whole-brain, 3D images. Moreover, to further evaluate and advance the clinical utility of the resulting N^3^ biomarker, its relation to genetic, environmental, and other clinical risk factors ^55–58^ needs to be robustly investigated. Finally, the current implementation of N^3^ limits spatial interpretability regarding the anatomical locus of detected abnormalities. Future extensions should incorporate regional granularity through region-of-interest (ROI) based analyses, applying the N^3^ framework to parcellated brain volumes or adding ROIs as an additional stratification dimension, thereby capturing region-specific aging trajectories and spatial vulnerability patterns. Generally, adding model-agnostic interpretability methods such as Shapley values would transform N^3^ into a feature-resolved normativity mapping framework.

Finally, the effectiveness of the N^3^ framework is dependent on the choice and robustness of the local density estimation strategy. In our application, the Nearest Neighbor Algorithm depends on the *k* parameter, which defines the number of neighbors considered in the quantification. Limiting the number of neighbors to 10% with an upper bound of 15 prevented overly broad comparisons while maintaining sufficient robustness across all control groups. Sensitivity analysis demonstrates robust N^3^ performance across several variations in *k* values, with increasing *k* systematically trading precision for recall (see Supplementary Information Table S1, 2). When applied to novel scenarios, the underlying algorithm should be customized or adapted to the unique requirements, e.g., by optimizing the nearest neighbor parameter *k*, choosing other local density estimation algorithms, using custom distance metrics, or leveraging dimensionality reduction techniques^35,59^.

Conceptually, our work contributes to the field by redefining normativity as a landscape of physiologically permissible configurations rather than proximity to a single group-average prototype. By demonstrating that such diversity-aware normativity concepts can be translated into an effective biomarker of brain structure, our results provide a concrete pathway for moving from population-level averages toward individualized, configuration-based characterization of neurodegenerative and potentially other medical conditions.

## Conclusion

The insights presented in this work show the potential of reevaluating how population norms are derived, applied, and interpreted in clinical practice. With our N^3^ framework, we operationalize normativity as a multidimensional, continuous landscape that emerges from the diverse configurations observed in a reference population. Applied to brain structure, the N^3^ biomarker was able to distinguish biologically coherent variants from truly atypical constellations and improved the detection of neurodegenerative diseases both in statistical as in well as in single-subject predictions. As we continue to refine and validate our N^3^ framework, it is our hope that the insights and ideas presented here will contribute to more diversity-aware normativity assessments and support our collective scientific endeavors to realize truly individualized patient care.

## Supplementary information

Transparent Peer Review file

Supplementary Information

## Data Availability

The source data for Fig. 5b is provided in Supplementary Table S5. Data were obtained from the German National Cohort (NAKO), the Alzheimer’s Disease Neuroimaging Initiative (ADNI), the Open Access Series of Imaging Studies 3 (OASIS3), the Frontotemporal Lobar Degeneration Neuroimaging Initiative (NIFD, and the Australian Imaging, Biomarker & Lifestyle Study of Aging (AIBL).The data of the MACS study, a DFG-funded ongoing cohort study conducted at the University of Münster and the University of Marburg, are not publicly available. Access to the data is restricted due to the ongoing nature of the research project and data protection obligations governing participant confidentiality. All other data are available upon request via the access management systems of the respective studies. (NAKO: nako.de/forschung, ADNI, AIBL, NIFD: ida.loni.usc.edu, OASIS3: sites.wustl.edu/oasisbrains)

## Code availability

Code to realize the normativity estimation calculations within the N^3^ framework is written in the Python programming language and is provided as an open-source resource to the scientific community on Github (https://github.com/RLeenings/n3_framework)^60^.
