# Supplementary material for: Contextualizing biological heterogeneity in brain structural normativity assessments across the aging continuum with the nearest neighbor normativity framework
Source: Commun Med (Lond). 2026 Nov 13;6:587. doi: 10.1038/s43856-026-01903-0 (PMC13768337; doi:10.1038/s43856-026-01903-0)
Supplement: Supplementary file 1 [file 43856_2026_1903_MOESM1_ESM.pdf]

nature portfolio

Peer Review File

Contextualizing Biological Heterogeneity in Brain Structural Normativity Assessments Across the Aging Continuum with the Nearest Neighbor Normativity Framework

Corresponding Author: Professor Ramona Leenings

This file contains all reviewer reports in order by version, followed by all author rebuttals in order by version.

Version 0:

Reviewer comments:

Reviewer #1

(Remarks to the Author)
In this manuscript, the authors propose a novel methodology to extract brain imaging biomarkers for neurodegenerative disorders, addressing the limitations of current approaches that rely on population averages. They leverage a large dataset to demonstrate the statistical advantages of the proposed method compared with normative modeling and the brain age approach. The manuscript is well-written; however, several issues need to be addressed before it is suitable for publication.

Major:

The description of Figure 2 is somewhat vague. It would be helpful to specify which “brain structure” was used to generate this figure. Additionally, the statement about the green line could be clarified—for example, “The individual’s chronological age is depicted in green” or “Chronological age is depicted in green”. The term “level of coherence” is also unclear; does it refer to the “similarity of the brain feature between the individual and the age-matched control group”? Clarifying these points would improve the figure legend’s clarity.

At line 287, some of the mathematical notations are misleading. By “Xc $\in$ X”, I believe the authors mean that Xc is a subsample (i.e., a selection of rows) of X. The notation $x \in X$ usually implies set membership, not “vector is a column (or row) in a matrix.” The matrix X in the manuscript is not a set, mathematically. Similar issues occur with other notations, such as “Nqi $\subseteq$Xc” and “x′ $\in$Xc”. A better way to notify a vector x is a row of a matrix X is the following latex:

```
\[
X \in \mathbb{R}^{n \times m}, \quad x = X_{i,:}, \quad i \in \{1, \dots, n\}.
\]
```

Also, in equation 1, it is not proper to use: min({a|a $\in$ Aj }). If the authors intend to indicate the minimum value of the j-th column of the matrix A, it is sufficient to write: min(A_j), or more explicitly, with the following latex:

```
\[
\min_{i} A_{ij}
\]
```

At line 516, the authors mention they aggregate z-scores into two normative modeling markers with the sum of absolute z-scores and the count of absolute z-scores > 1.96. It’s better to make it clear that they “sum the absolute z-scores over the features to get a total score for each sample”. More importantly, it’s necessary to explain why they sum the absolute z-scores. This will make the highest and lowest values very close. I believe it’s better to use PCA or more sophisticated dimension reduction methods such as t-SNE or UMAP.

On page 20, the authors mention that they use a machine learning method (SVM) to evaluate the effectiveness of aging

markers in classifying neurodegenerative diseases. It is necessary to make it clear what the features are fed into to SVM model for the NM-C, NM-S, BAG, and N3 (The markers shown in Table 1).

In Figure 4 (page 11), the authors report a negative correlation between BAG and age. This is a well-known statistical phenomenon in regression models. As mentioned, the authors applied bias correction in age prediction; ideally, this negative correlation should be removed after correction. It is unclear whether the authors estimated the bias-correction model using the training data and then applied it to the test data. Personally, I believe it is acceptable to use the test data to estimate the bias-correction model so that the corrected BAG is orthogonal to age—effectively regressing out chronological age—which is a common practice in linear models.

Finally, the authors do not mention which brain atlas was used to extract imaging features such as GM, WM, WMH, etc. Did they use whole-brain values? This approach is uncommon, as it overlooks regional structural variations and the distinct developmental trajectories of different brain areas. It's also important to show how the proposed method performs on data with high dimensionality.

Minor:

Typo in the description of Figure 1 on page 4: "It ca (can) be adapted to a variety of clinical inquiries…"

At line 182, ρ=0.21 (should be -.021?)

At line 331, it is better to clarify how the different control groups are identified and why they are designed not to be mutually exclusive. Are they identified simply by age and sex (as mentioned at line 381)?

On page 22, the link to GitHub for the code resource is not provided.

Reviewer #2

(Remarks to the Author)
This manuscript proposes an alternative approach for normative modeling (nearest neighbor normativity, or N3). By dividing a normative population into demographically matched subgroups, it aims to address the limitations of alternative formulations. Mainly, the reliance of standard normative modeling on generating a single, average normative distribution, and the dependence of the brain-age gap (BAG) on summarizing aging trajectories into a single scalar: chronological age. The authors propose dividing the normative population into 100 sex- and age-matched control groups constructed using k-nearest-neighbor clustering based on the Euclidean distance metric. For each study participant, his/her brain representation is composed of its gray matter, white matter, hyperintense white matter, total intracranial, and cerebrospinal fluid volumes. Local densities are estimated within each control group, and a corresponding density function is fitted. The likelihood of a given sample belonging to each group is then computed to form a normativity-profile vector, which is subsequently collapsed into a single scalar measure referred to as the N3 coefficient.
Overall, I think the approach is interesting and it does address some of the limitations of BAG and standard normative strategies. However, I think this manuscript needs to address some issues prior to being considered for publication.

Clarity of N3 computation
- First of all, the explanation of how the N3 coefficient is calculated is not clear. It is key for the manuscript to provide a clearer description of this key step of the proposed formulation.
Biological interpretability
- The paper refers to the different estimated metrics (e.g., BAG, N3) as biomarkers. Strictly speaking they are as they are derived from brain images. However, I tend to think of biomarkers as indicators of a biological state or the health of a person. Taking that perspective into consideration, what is it that the N3 coefficient can tell us about the overall health (or aging process) of a person? While predictive performance is key in health applications, biological interpretability can be just as important. Maybe intermediate brain representations could be estimated on top of the proposed approach?
- While whole volume metrics can provide a sense of brain structure, this is limited by the fact that these are global metrics, i.e., they do not analyze local patterns or even the location of potential abnormalities. Again, what is it that the proposed methodology can tell us of the health of an individual's brain?
Appropriateness of training data set
- Is the training set representative of the general population? I bring this up because it seems to be composed solely of brain images from the German National Cohort, even though the authors have used data from other studies which do have healthy controls in them, albeit in smaller numbers. It would be good to have improved diversity in it by incorporating scans from other studies too.

Reviewer #3

(Remarks to the Author)
The introduction provides a clear and compelling motivation for the study and effectively positions the proposed framework within the broader neuroimaging literature. However, several aspects could be improved to enhance clarity and focus. The section is somewhat overloaded with methodological detail — the four-step outline of the N3 framework reads more like part of the Methods than an introduction, and a more concise overview would improve readability. In addition, the narrative

occasionally blurs the line between background and findings, particularly in statements that imply results before they are presented. The text also relies heavily on technical terminology and dense phrasing, which may limit accessibility for a broader multidisciplinary audience. Expanding the conceptual discussion of “normativity” and inter-individual variability would further strengthen the intellectual grounding of the work.

The Results section effectively demonstrates the value of the N3 framework, presenting robust analyses that show its superiority over traditional normative modeling and Brain Age methods. The inclusion of multiple evaluation metrics and validation steps enhances the credibility of the findings. However, the section would benefit from clearer focus and organization. Several passages contain excessive technical detail, which obscures key takeaways and makes the results harder to interpret. Streamlining descriptions of model comparisons and statistical tests would improve readability. The authors should also clarify the clinical relevance of the reported performance gains — particularly for conditions such as Mild Cognitive Impairment, where the advantage of N3 appears modest. Additionally, some interpretive statements drift into discussion, and moving these to the Discussion section would help maintain a sharper distinction between results and interpretation. Summarizing key findings at the end of each subsection could help reinforce the main messages and guide the reader through the dense analyses.

The Discussion effectively highlights the conceptual contribution of the N3 framework and situates it within the broader goals of precision medicine. The authors clearly articulate how N3 advances beyond population-average models and link their findings to practical implications in neurodegenerative disease research and early diagnosis. To improve, the Discussion should streamline its messaging and trim details already covered in previous sections, allowing greater focus on interpretation and conceptual insight. The authors should further emphasize the theoretical and clinical significance of their findings — for example, how N3 might reshape normative modeling practices or inform individualized treatment strategies. While the study’s limitations are acknowledged, their practical impact on smaller clinical studies and potential strategies for mitigation deserve deeper exploration. The section would also benefit from a clearer synthesis of how the results advance understanding in the field and from outlining concrete next steps for research and clinical translation.

The Conclusion succinctly summarizes the core contribution of the study and reiterates its potential relevance to personalized medicine. It effectively reinforces the translational promise of the N3 framework across medical domains. The authors could more explicitly link the closing statements to the key empirical findings and highlight what the study concretely adds to existing knowledge. They should also clarify the specific next steps for validation or clinical implementation, as the current phrasing remains largely aspirational. Finally, tightening the language to avoid repetition (e.g., “personalized healthcare” and “personalized patient care”) would improve clarity and impact.

Methods
-Major Concerns:
Conceptual clarity and reproducibility: The description of the N3 algorithm is mathematically comprehensive but would benefit from clearer organization. Presenting the conceptual framework separately from the implementation details (e.g., the neuroimaging case) would make the methods easier to navigate and improve reproducibility.

Equation (2) – Density estimation: The formula does not align with standard local density estimation practices and lacks normalization by dimensionality or the number of neighbors (k). A justification for this formulation should be provided.

Equation (5) – Exponentiated Weibull distribution: The distribution’s mathematical form appears incomplete (missing a scale parameter), and parameter estimation procedures are not described. The rationale for choosing this distribution for λ′ values should be clarified.

Equation (6) – Signed likelihood rule: The conditional “if $x < 1$” seems arbitrary after normalization and should instead compare against the median value to correctly determine direction of deviation.

Parameter k selection: The fixed choice of $k = \min(\text{round}(0.1 \times n), 15)$ is arbitrary and not empirically justified. A sensitivity analysis or rationale for this threshold should be included.

Meta-normativity layer (Eq. 9): The second-layer estimation step is conceptually under-specified. The authors should clarify what distance metric and normalization procedures are used when comparing normativity profiles and how independence between layers is maintained.

-Minor Concerns:

Inconsistent notation (e.g., alternating between q, x, and p for samples, and between ϕ and φ for normativity profiles).

Typographical error (“medium” should read “median”).

The term “control group” is used variably to mean both a demographic subgroup and a reference sample — clearer terminology would help.

Merging adjacent age bins in Section 5.1.5 could introduce bias or information leakage across strata and should be statistically justified.

The inference-phase description (lines 370–379) lacks detail on how stored parameters from the second layer are reused for

new samples.

Figures

- Figure 1 and Figure 2:

Including both figures in the Introduction is unconventional; they primarily describe the algorithmic framework and would be better placed in the Methods or Supplementary Material.

Figure 1’s depiction of “normativity profiles” largely repeats the text; the visual should emphasize workflow, data flow, or hierarchical structure to add distinct value.

Figure 2’s example profiles (three individuals) require labeled axes and a clear legend clarifying what “alignment” represents and how to interpret deviations.

- Figure 3:

The top panel presents effect sizes (η^2), but the axes, units, and color scheme are unclear — axis labels and a complete legend should be added.

The bottom panel includes multiple metrics (balanced accuracy, F1-score, recall, precision) that make the plot crowded; highlighting the main metric or separating panels could improve readability.

Captions should remain descriptive — current phrasing mixes figure content with interpretive commentary (e.g., “larger explained variance”).

- Figure 4:

The ICC analysis is relevant, but subplots (a–c) should be explicitly labeled and referenced in the caption for clarity.

Replace subjective phrasing such as “converges to excellent levels” with quantitative language (e.g., “ICC ≥ 0.9 for n ≥ 300”).

- Table 1:

The table effectively summarizes statistical and machine-learning results but is dense; splitting it into two (statistical vs. predictive metrics) or moving detailed values to Supplementary Material would enhance readability.

Ensure consistent bolding of best-performing results and define all abbreviations (NM-S, NM-C, BAG, N^3) directly in the caption.

- Table 2:

The dataset summary is valuable but could be formatted for easier reading (aligned columns, consistent decimal places).

Add brief notes on inclusion/exclusion criteria and clarify whether participants contributed multiple scans to improve transparency.

Version 1:

Reviewer comments:

Reviewer #1

(Remarks to the Author)
The authors have thoroughly addressed the points raised in the initial review. Specifically, the additional clarifications and formula corrections in the Methodology and Results section have strengthened the manuscript's findings. I am satisfied with the current version and believe it now meets the standards for publication. I recommend acceptance.

Reviewer #2

(Remarks to the Author)
I appreciate the authors' efforts in clarifying the mathematical framework of the N3 algorithm. The revised methods section has significantly improved the explanation of this approach.

While the model remains limited by its reliance on global volumetric measures rather than local spatial patterns, the authors have successfully argued that N3 is intended to capture how multivariate volumetric patterns of a particular individual correlate with those in different aging subgroups, which would be missed by univariate or standard normative strategies. I also thank the authors for incorporating this limitation in the discussion.

Reviewer #4

(Remarks to the Author)
I thank the Editor for the opportunity to evaluate the revised version of this manuscript and the accompanying rebuttal letter.

The authors have substantially revised the manuscript and addressed many of the concerns raised during the previous review round. The revised version is considerably clearer in its methodological presentation and in the conceptual framing of the proposed Nearest Neighbor Normativity (N^3) framework.

Overall, I find the work interesting and potentially relevant for the neuroimaging and computational neuroscience communities. The manuscript addresses an important and timely issue, namely the limitations of population-average approaches for modeling heterogeneous brain aging trajectories and individualized norm deviations. The proposed framework introduces a contextualized and density-based perspective on normativity assessment that differs conceptually from both classical normative modeling and brain-age paradigms.

One of the main strengths of the manuscript is that it reframes normativity not as a deviation from a single average trajectory, but as a structured space of plausible configurations. I believe this conceptual perspective is novel and may stimulate further discussion in the field regarding the role of inter-individual variability in neuroimaging biomarkers and personalized medicine approaches.

The authors have also improved several aspects of the manuscript in response to the reviewers' comments, including:

-clarification of the N^3 computation and notation,
-better explanation of the machine learning evaluation setup,
-expanded discussion of biological interpretability,
-additional analyses for normative modeling aggregation strategies,
-and clarification of the BAG bias correction procedure.

The manuscript is now substantially more understandable and methodologically transparent than the previous version.

Nevertheless, I believe some important limitations should still be more explicitly acknowledged and discussed.

First, while the manuscript conceptually emphasizes "multiple normative prototypes" and a "landscape of plausible configurations", the current implementation remains largely based on demographic subgrouping strategies (primarily age- and sex-matched local neighborhoods). As currently formulated, the plurality of normative states appears to arise mainly from localized demographic contextualization rather than from the identification of biologically distinct latent trajectories. I therefore encourage the authors to slightly moderate some conceptual claims and clarify this distinction more explicitly.

Second, although the framework is presented as broadly applicable to complex neurobiological representations, the current validation relies on a relatively low-dimensional set of global volumetric measures (GM, WM, WMH, CSF, TIV). The authors appropriately acknowledge this limitation in the revised Discussion. However, I believe the manuscript would benefit from a clearer statement that the current study primarily demonstrates the conceptual feasibility of the framework rather than a definitive validation in genuinely high-dimensional neuroimaging settings.

Third, while the methodological description has improved substantially, some details regarding subgroup construction and overlap remain somewhat difficult to fully reconstruct. Additional clarification regarding subgroup overlap, neighborhood definition, subgroup size selection, and statistical dependence between subgroups would further improve reproducibility.

Regarding statistical validity, the analyses appear generally appropriate and carefully conducted. I particularly appreciate the additional clarification regarding the BAG bias correction strategy and the inclusion of multiple normative modeling aggregation approaches. The comparative evaluation across statistical effect sizes and single-feature classification analyses is coherent with the stated goals of the study.

Overall, I believe this revised manuscript represents a thoughtful and conceptually interesting contribution to the discussion surrounding personalized normative assessment in neuroimaging. While some conceptual claims may still benefit from slightly more cautious framing, I believe the manuscript has been substantially strengthened through revision and is close to being suitable for publication after relatively minor clarifications.

Version 2:

Reviewer comments:

Reviewer #4

(Remarks to the Author)
Overall, I believe the authors have carefully addressed the concerns raised during the previous review rounds and have substantially strengthened the manuscript. The methodological description is now considerably clearer, the limitations of the

current proof-of-concept study are more appropriately acknowledged, and the conceptual framing of the proposed N^3 framework has been improved.

I therefore support publication of the manuscript in its current form.

Open Access This Peer Review File is licensed under a Creative Commons Attribution 4.0 International License, which permits use, sharing, adaptation, distribution and reproduction in any medium or format, as long as you give appropriate credit to the original author(s) and the source, provide a link to the Creative Commons license, and indicate if changes were made.
In cases where reviewers are anonymous, credit should be given to 'Anonymous Referee' and the source.
The images or other third party material in this Peer Review File are included in the article’s Creative Commons license, unless indicated otherwise in a credit line to the material. If material is not included in the article’s Creative Commons license and your intended use is not permitted by statutory regulation or exceeds the permitted use, you will need to obtain permission directly from the copyright holder.
To view a copy of this license, visit https://creativecommons.org/licenses/by/4.0/

RESPONSE LETTER

Judged by your neighbors: A novel framework for personalized assessment of brain structural aging effects in diverse populations

COMMSMED-25-2286-T

We would like to express our great thankfulness to the reviewers for their valuable remarks, which helped us to substantially improve the quality and clarity of the manuscript.

Reviewer #1 (Remarks to the Author):

In this manuscript, the authors propose a novel methodology to extract brain imaging biomarkers for neurodegenerative disorders, addressing the limitations of current approaches that rely on population averages. They leverage a large dataset to demonstrate the statistical advantages of the proposed method compared with normative modeling and the brain age approach. The manuscript is well-written; however, several issues need to be addressed before it is suitable for publication.

Major:

The description of Figure 2 is somewhat vague. It would be helpful to specify which "brain structure" was used to generate this figure. Additionally, the statement about the green line could be clarified—for example, "The individual's chronological age is depicted in green" or "Chronological age is depicted in green". The term "level of coherence" is also unclear; does it refer to the "similarity of the brain feature between the individual and the age-matched control group"? Clarifying these points would improve the figure legend's clarity.

We thank the reviewer for carefully pointing out these ambiguities in the figure legend. We have adapted the figure description in alignment with the suggestions.

Fig. 1: "For illustration, we show the normativity profiles of three individuals of the training sample. The N^3 framework compares an individual's brain structural features (global volumes of gray matter, white matter, white matter hyperintensities, cerebrospinal fluid and total intracranial volume) to those seen in different age groups along the age continuum. The normativity profile shows the local sample density in different age groups, i.e. how common similar volumetric configurations appear along the aging continuum (blue) as well as the individual's chronological age (green). (a) An individual's data shows higher local sample density in younger age groups, indicating that the volumetric configuration is more common within younger age groups and less common within same-aged individuals (b) An individual's volumetric configuration appears more common within older age groups, indicating premature aging effects. (c) A brain structure appears most common within its own age group and deprecating occurrence within other age groups."

At line 287, some of the mathematical notations are misleading. By "Xc ∈ X", I believe the authors mean that Xc is a subsample (i.e., a selection of rows) of X. The notation $x \in X$ usually implies set membership, not "vector is a column (or row) in a matrix." The matrix X in the manuscript is not a set, mathematically. Similar issues occur with other notations, such

as “Nqi ⊆Xc” and “x′∈Xc”. A better way to notify a vector x is a row of a matrix X is the following latex:

\[
X \in \mathbb{R}^{n \times m}, \quad x = X_{i,:}, \quad i \in \{1, \dots, n\}.
\]

Also, in equation 1, it is not proper to use: min({a|a∈Aj }). If the authors intend to indicate the minimum value of the j-th column of the matrix A, it is sufficient to write: min(A_j), or more explicitly, with the following latex:

\[
\min_{i} A_{ij}
\]

We thank the reviewer for pointing out the notation issues and for the correction. We have integrated the proposed correction into the section and overall, extensively revised the N³ framework description to increase understandability. Please see the revised Methods section on page 14 and the snippet given below.

Methods: *The algorithm quantifies the local sample density in different subgroups, defined as submatrixes* $X_c \in \mathbb{R}^{n \times m}, c = 1, 2, \ldots, g$ *of a reference dataset* $X \in \mathbb{R}^{a \times b}$. *We normalize each submatrix* X_c *column-wise using min–max scaling so that all entries satisfy* $X_{cij} \in [0, 1]$ *for* $i = 1, 2, \ldots n, j = 1, 2, \ldots m$

Within each subgroup, we use a Nearest Neighbor approach and define a subset N *for each sample* q *such that it contains the* k *points in* X_c *which are the closest to sample* q. *Distance* d *is measured using the Euclidean distance. We define the local sample density* $\lambda(q, c)$ *of sample* q *in subgroup* c *as the inverse of the average distance to its* k *nearest neighbors.*

$$\lambda(q_i, c) = (\frac{1}{k}\sum_{j=1}^{k}\|x_j - q\|)^{-1}$$

…

At line 516, the authors mention they aggregate z-scores into two normative modeling markers with the sum of absolute z-scores and the count of absolute z-scores > 1.96. It’s better to make it clear that they “sum the absolute z-scores over the features to get a total score for each sample”. More importantly, it’s necessary to explain why they sum the absolute z-scores. This will make the highest and lowest values very close. I believe it’s better to use PCA or more sophisticated dimension reduction methods such as t-SNE or UMAP.

We thank the reviewer for this important and concrete suggestion and have revised the text according to the suggestions. We clarify that our intention was to use a simple, transparent aggregation that directly reflects the overall distance from the population average across all features. However, we fully agree with the provided suggestions and have complemented our evaluation by adding averaging and a UMAP-based non-linear embedding as aggregation strategies. The results show that no single aggregation strategy dominates across all criteria and that the different aggregation strategies capture partially distinct aspects of norm deviations, with the outlier-count marker emerging as the most robust and interpretable summary in our setting. We provide the results in the Supplementary Information and, for simplicity, discuss the outlier-count normative modeling marker in the manuscript.

Methods: *We calculate normative models on the training data using the Predictive Clinical Neuroscience toolkit as described in Rutherford et al.. To train the models, we normalize GM, WM, WMH, CSF by Total Intracranial Volume (TIV) and fit Bayesian Linear Regression models with default parameters. Subsequently, we derive z-scores for each variable and aggregate them into four normative modeling markers, each yielding a single score per individual. The best performing marker counts the number of absolute z-scores greater than 1.96, reflecting the burden of outlying features per person, and is discussed throughout the manuscript. We evaluated three more aggregation strategies. The second marker sums the absolute z-scores across all features, capturing the overall magnitude of deviation from the normative reference. The third marker is defined as the average z-score, summarizing the mean direction and extent of deviation. Finally, we obtain a low-dimensional embedding of the multivariate z-score patterns using a UMAP component, which serves as a fourth, non-linear aggregation of normative deviations. For completeness, Table C4 and C5 in Appendix reports the statistical and machine-learning performance of all evaluated markers.*

Supplementary Information*: We evaluated different aggregation strategies of the Normative Modeling z-scores. The tables summarize effect sizes from group-level statistics and predictive performance from machine learning analyses, allowing a direct comparison of how each aggregation strategy supports disease classification. Aggregations based on the overall count of outliers and the absolute sum of deviations yielded consistently larger effect sizes in the statistical analyses, whereas averaging and the UMAP-based aggregation of z-scores resulted in negligible effects for MCI and AD. In contrast, machine learning classifiers benefited from UMAP-based aggregation, particularly in FTD, and from averaging in MCI, indicating that these strategies encode informative structure that can be exploited by non-linear models for case-level prediction. Overall, different diseases benefit from different aggregation strategies, while the count of outliers emerges as a robust choice for both statistical and machine learning analyses across diseases.*

On page 20, the authors mention that they use a machine learning method (SVM) to evaluate the effectiveness of aging markers in classifying neurodegenerative diseases. It is necessary to make it clear what the features are fed into to SVM model for the NM-C, NM-S, BAG, and N3 (The markers shown in Table 1).

We apologize for the earlier unclear formulation. The goal with this analysis was to assess how well each algorithm captures norm deviations that may be indicative of disease. We therefore use each biomarker as the sole feature to train a classifier and then quantify its predictive value. This single-feature design isolates each biomarker's intrinsic discriminative capacity, ensuring that performance differences reflect the information content of the biomarker itself rather than synergistic effects with additional features or confounding by classifier architecture. We selected Support Vector Machines with an *rbf* kernel because they can capture non-linear decision boundaries even in single-feature scenarios, which is appropriate for biomarkers that may exhibit non-monotonic relationships with disease. We have clarified this procedure in both the Results and Methods sections.
Results: *"Second, we conduct machine learning analyses to evaluate each biomarker's ability to predict the occurrence of a neurodegenerative disease on a single-subject level. To this end, we train non-linear models using each biomarker as a single input feature, enabling us to quantify the degree to which each biomarker captures disease signals in the data. "*
Methods: *"Models were trained with each of the biomarkers as the only feature, enabling us to quantify each biomarker's ability to capture norm deviations indicative of neurodegenerative diseases. "*

In Figure 4 (page 11), the authors report a negative correlation between BAG and age. This is a well-known statistical phenomenon in regression models. As mentioned, the authors applied bias correction in age prediction; ideally, this negative correlation should be removed after correction. It is unclear whether the authors estimated the bias-correction model using the training data and then applied it to the test data. Personally, I believe it is acceptable to use the test data to estimate the bias-correction model so that the corrected BAG is orthogonal to age—effectively regressing out chronological age—which is a common practice in linear models.

We thank the reviewer for this important remark and apologize for the lack of clarity. Following de Lange & Cole (2020) and Peng et al. (2021), we estimated bias-correction parameters on a held-out validation set (n~3000, 10% of normative data, age-stratified). We apply this correction model to all test data before computing BAG. We have clarified our procedure in the methods section. The residual correlation in Figure 4 ($\rho = -0.21$) reflects test-set performance. As age is included as covariate in the statistical analyses, any residual age bias should be catched. Chronological age is additionally included as a covariate in all downstream statistical analyses, so any residual association between BAG and age should be further accounted for at the statistical modeling stage.

Lange, A.-M. G. de & Cole, J. H. Commentary: Correction procedures in brain-age prediction. NeuroImage: Clin. ***26****, 102229 (2020).*
Peng, H., Gong, W., Beckmann, C. F., Vedaldi, A. & Smith, S. M. Accurate brain age prediction with lightweight deep neural networks. Méd. Image Anal. 68, 101871 (2021).

Methods: *We use age-stratified 90% of the available normative dataset for model training. We use the remaining 10% of the normative dataset to train a linear age bias correction as described in Peng et al.. For the evaluation of unseen samples, we use the Brain Age SVM model to predict age and subsequently apply the bias correction model, before we calculate the difference between the chronological and predicted age, the BAG.*

Finally, the authors do not mention which brain atlas was used to extract imaging features such as GM, WM, WMH, etc. Did they use whole-brain values? This approach is uncommon, as it overlooks regional structural variations and the distinct developmental trajectories of different brain areas. It's also important to show how the proposed method performs on data with high dimensionality.

We thank the reviewer for raising this important point. In the revised manuscript, we now clarify that GM, WM, WMH, CSF, and TIV were derived as tissue volumes from the whole-brain image using the CAT12-based tissue segmentation procedure. We fully acknowledge that global features aggregate heterogeneous regional processes and lack spatial specificity. This is a substantive limitation for clinical research as neurodegenerative diseases exhibit region-specific vulnerability and our features cannot spatially localize detected abnormalities. However, the demonstrated performance improvements even at this global level support the framework's core principle, which is that contextualized processing and multi-prototype approaches enhance sensitivity compared to population-average approaches. We are extending N^3 to regional parcellation features and to whole-brain, high-dimensional embeddings obtained from self- or weakly supervised deep learning and medical foundation models, in order to systematically evaluate its performance in genuinely high-dimensional settings. We have clarified this limitation in the discussion section.

Discussion: *In our evaluation, the N^3 approach has demonstrated its ability to effectively decode the information contained in these limited neurophysiological variables and was able to extract more meaningful insights compared to conventional approaches. However, in future work, the N^3 framework needs to replicate these results for example in the processing of high-dimensional data. To this end, deep-learning based image representation could be used to evaluate the efficacy of the N^3 framework in parsing whole-brain, 3D images. Moreover, to further evaluate and advance the clinical utility of the resulting N^3 biomarker, its relation to genetic, environmental and other clinical risk factors needs to be robustly invested .*

Minor:

Typo in the description of Figure 1 on page 4: "It ca (can) be adapted to a variety of clinical inquiries…"

At line 182, ρ=0.21 (should be -.021?)
We thank the reviewer for noticing these mistakes and have corrected both.

At line 331, it is better to clarify how the different control groups are identified and why they are designed not to be mutually exclusive. Are they identified simply by age and sex (as mentioned at line 381)?

We thank the reviewer for identifying this ambiguity. We have clarified the terminology throughout: we now use "subgroups" to refer to the algorithmic framework and "demographic subgroups" for our specific neuroimaging implementation. Generally, the framework allows to evaluate normativity from different viewpoints, which might lead to subgroups not being mutually exclusive. This would for example be the case if one subgroup is defined as individuals with severe symptoms, and another subgroup is defined as individuals with a particular metabolic profile, and some individuals are fulfilling both criteria, therefore being representative for both clinical populations and thus part of both subgroups.

On page 22, the link to GitHub for the code resource is not provided.
We apologize for this oversight and have added the missing link

Reviewer #2 (Remarks to the Author):

This manuscript proposes an alternative approach for normative modeling (nearest neighbor normativity, or N3). By dividing a normative population into demographically matched subgroups, it aims to address the limitations of alternative formulations. Mainly, the reliance of standard normative modeling on generating a single, average normative distribution, and the dependence of the brain-age gap (BAG) on summarizing aging trajectories into a single scalar: chronological age.
The authors propose dividing the normative population into 100 sex- and age-matched control groups constructed using k-nearest-neighbor clustering based on the Euclidean distance metric. For each study participant, his/her brain representation is composed of its gray matter, white matter, hyperintense white matter, total intracranial, and cerebrospinal fluid volumes. Local densities are estimated within each control group, and a corresponding density function is fitted. The likelihood of a given sample belonging to each group is then computed to form a normativity-profile vector, which is subsequently collapsed into a single scalar measure referred to as the N3 coefficient.
Overall, I think the approach is interesting and it does address some of the limitations of BAG and standard normative strategies. However, I think this manuscript needs to address some issues prior to being considered for publication.

Clarity of N3 computation
- First of all, the explanation of how the N3 coefficient is calculated is not clear. It is key for the manuscript to provide a clearer description of this key step of the proposed formulation.

We thank the reviewer for pointing out this important shortcoming. We have extensively revised the algorithm description, in the Result section, as well as in the Method section in both text and mathematical formulation.

Biological interpretability
- The paper refers to the different estimated metrics (e.g., BAG, N3) as biomarkers. Strictly speaking they are as they are derived from brain images. However, I tend to think of biomarkers as indicators of a biological state or the health of a person. Taking that perspective into consideration, what is it that the N3 coefficient can tell us about the overall health (or aging process) of a person? While predictive performance is key in health applications, biological interpretability can be just as important. Maybe intermediate brain representations could be estimated on top of the proposed approach?

We sincerely thank the reviewer for this conceptually important distinction and for prompting us to clearly describe what the N³ biomarker represents from a biological and clinical interpretability perspective. This feedback has substantially improved our framing in the results and discussion sections. We believe this perspective substantially strengthens the interpretability of our approach.

Results: *In a first step, the N³ framework analyzes the frequency of different brain structural configurations within sex-matched subgroups of successive chronological age. The result is a vector that expresses how commonly an individual's specific configuration occurs within each demographic subgroup, i.e. how their measurements are embedded within the natural variety seen along the aging continuum. We call this vector a normativity profile. Its shape indicates how an individual's configuration is positioned relative to expected trajectories of brain structural decline. For example, an individual's configuration may show similarity with those occurring frequently in younger age groups, indicating younger brain features and fewer aging effects as commonly seen in same-aged individual (see Fig. \ref{fig:profiles}). However, normativity profiles that shift away from an individual's chronological age are not necessarily indicative of disease. Since genetic and environmental influences on brain structure accumulate over time, we expect substantial variability in normativity profile shapes, particularly in older age groups. We therefore quantify how frequently different normativity profile shapes occur within each age group, thereby allowing for several normative variants of brain structural decline. In a second step, the N³ framework therefore assesses how common a given profile shape is within each sex- and age-specific subgroup. The resulting metric is the N³ biomarker, which provides an individualized index of how typical or atypical a person's brain structural configuration appears relative to the variability observed in both neurophysiology and aging trajectories across individuals.*

Discussion: *In the application of the N³ framework to brain structure, we chose demographic subgroups as the primary context for interpretation. In this setting, the algorithm quantifies how common a particular combination of global brain volumes is across the diversity of brain structural measurements observed among same-sexed individuals along the aging continuum. The resulting vector, which we refer to as the normativity profile, encodes the resemblance of an individual's brain to different natural atrophy progression states for that sex across the lifespan. In the second step, we contextualize each normativity profile against those of same-aged individuals to determine how frequently a given profile shape occurs within that age group. The resulting biomarker quantifies the typicality of an individual's aging pattern among their peers. In clinical applications, the same procedure could be adapted to alternative contextualizations, such as profiles typical for specific comorbidity constellations or genetic or environmental risks.*
Because N³ relies on local density estimation, it can represent similar aging patterns in neighboring age groups without forcing a trade-off in normativity metrics between them. More specifically, a particular configuration can be equally typical in several adjacent age groups. This contrasts with brain age models, which must assign a single best-fitting age and compress the richness of aging trajectories into a point estimate. Moreover, the N³ framework is able to detect irregular configurations that are uncommon across all age groups, and differentiate between general irregular anatomy and age-inappropriate configurations. Finally, the N³ framework is through its conceptualization able to revealing potential shifts towards constrained and pathologically similar configurations, if disease erodes the natural spectrum of variability and converts uniqueness into uniform manifestations.

- While whole volume metrics can provide a sense of brain structure, this is limited by the fact that these are global metrics, i.e., they do not analyze local patterns or even the location of potential abnormalities. Again, what is it that the proposed methodology can tell us of the health of an individual's brain?

We appreciate the reviewer raising this important limitation regarding spatial interpretability. The N³ biomarker in our work quantifies how atypical an individual's multidimensional brain volumetric configuration appears. Specifically, it identifies whether the joint pattern of global tissue metrics deviates from expected patterns of whole-brain structural integrity. We agree with the reviewer and fully acknowledge that these global measurements cannot identify which specific brain regions are abnormal, which is a critical limitation for mechanistic understanding. We have addressed this limitation and expanded the discussion section:

Discussion: Finally, the current implementation of N³ limits spatial interpretability regarding the anatomical locus of detected abnormalities. Future extensions should incorporate regional granularity through region-of-interest (ROI) based analyses, applying the N³ framework to parcellated brain volumes or adding ROIs as an additional stratification dimension, thereby capturing region-specific aging trajectories and spatial vulnerability patterns. Generally, adding model-agnostic interpretability methods such as Shapley values would transform N³ into a feature-resolved normativity mapping framework.

Results:

Appropriateness of training data set
- Is the training set representative of the general population? I bring this up because it seems to be composed solely of brain images from the German National Cohort, even though the authors have used data from other studies which do have healthy controls in them, albeit in smaller numbers. It would be good to have improved diversity in it by incorporating scans from other studies too.

We thank the reviewer for raising this important point and fully agree that maximizing diversity in the reference data is critical. The German National Cohort (NAKO) is a large, nationwide, population-based study that recruited adults from 18 study centers across Germany using random sampling from local population registries. As such, it provides a broadly representative cross-section of the German adult population and a high-quality normative reference for structural brain imaging. At the same time, we acknowledge that NAKO is a national cohort and, like most study cohorts, most likely does not fully capture the full spectrum of clinical subgroups and underrepresented populations, which we now clarify as a limitation in the manuscript.
Moreover, our primary goal was to introduce and carefully characterize the N^3 framework. We therefore prioritized a clear assessment of the algorithm's unique contribution and deliberately used only the NAKO data as the reference set for model training, and treated all other cohorts as strictly external test sets. This design minimizes uncontrolled cohort- and scanner-specific distribution shifts within the normative reference data, which, in a Leave-One-Study-Out scenario, could affect the local density estimation and thus obscure the comparability of our results in-between the different studies and diseases. We fully acknowledge that, in a translational setting, it will be essential to extend the framework to more diverse, multi-site reference data, and to explicitly model and mitigate such distribution shifts. We have now clarified this point in the limitations.

Discussion: *In this initial proof-of-concept study, we used a multi-site, large-scale population cohort as a reference. While it is recruited to be representative, it is a national cohort and cannot reflect the entire spectrum of biological diversity. Future work should extend this approach to multi-cohort and internationally inclusive reference datasets in order to broaden generalizability and fairness across different populations. Moreover, to advance translation to clinical practice, it is essential to evaluate how effectively normative reference models derived from study cohorts can interpret data from clinical care routine.*

Reviewer #3 (Remarks to the Author):

The introduction provides a clear and compelling motivation for the study and effectively positions the proposed framework within the broader neuroimaging literature. However, several aspects could be improved to enhance clarity and focus. The section is somewhat overloaded with methodological detail — the four-step outline of the N3 framework reads more like part of the Methods than an introduction, and a more concise overview would improve readability. In addition, the narrative occasionally blurs the line between background and findings, particularly in statements that imply results before they are presented. The text also relies heavily on technical terminology and dense phrasing, which may limit accessibility for a broader multidisciplinary audience. Expanding the conceptual discussion of "normativity" and inter-individual variability would further strengthen the intellectual grounding of the work.

We thank the reviewer for this valuable feedback, which helped us to substantially clarify and refocus the introduction. In line with the suggestions, we have shortened and moved detailed descriptions of the N³ algorithmic steps to the methods section, carefully separated the background from any preview of findings and streamlined the algorithm description, including an extended conceptualization of normativity and inter-individuality, to improve accessibility for a broader multidisciplinary readership.

Introduction: *[...] To address the methodological shortcomings of the approaches described above, we propose a novel normativity estimation framework called Nearest Neighbor Normativity (N³).*

Normativity in our framework is defined as a structured space of permissible configurations that arises from the diverse physiological manifestations observed in healthy individuals. Healthy inter-individual variability reflects biologically principled movement in this space, whereas anomalies correspond to configurations that deviate in biologically unexpected directions. A biomarker should be able to distinguish physiologically coherent variants from truly atypical patterns that depart from the manifold of healthy configurations. Grounded within this conceptualization, the N³ framework shifts from estimating distance to an average, prototypical observation toward locating each individual within a high-dimensional landscape of plausible and implausible configurations.

In this work, we introduce the N³ framework and use it to assess the normativity of brain structures while explicitly accounting for the natural and systematic changes along the aging continuum. By relying on context-enriched assessments, the N³ is inherently designed to improve the understanding of variance in-between individual brain structures within the natural diversity seen along the aging continuum. We benchmark the resulting N³ biomarker relative to conventional neuroimaging biomarkers, including conventional normative modeling and Brain Age models. As a representative use case, we focus on neurodegenerative disorders, which are characterized by structural alterations and deviations from normative aging trajectories, and therefore provide a suitable testbed for the evaluation of the proposed framework.

The Results section effectively demonstrates the value of the N3 framework, presenting robust analyses that show its superiority over traditional normative modeling and Brain Age methods. The inclusion of multiple evaluation metrics and validation steps enhances the credibility of the findings. However, the section would benefit from clearer focus and organization. Several passages contain excessive technical detail, which obscures key takeaways and makes the results harder to interpret. Streamlining descriptions of model comparisons and statistical tests would improve readability. The authors should also clarify the clinical relevance of the reported performance gains — particularly for conditions such as Mild Cognitive Impairment, where the advantage of N3 appears modest. Additionally, some interpretive statements drift into discussion, and moving these to the Discussion section would help maintain a sharper distinction between results and interpretation. Summarizing key findings at the end of each subsection could help reinforce the main messages and guide the reader through the dense analyses.

We thank the reviewer for these thoughtful and constructive comments. In response, we have substantially revised the result section and improved its focus and organization. In particular, we have removed non-essential technical details, included a section introducing the biological interpretation of the N^3 biomarker and shifted interpretative and clinically oriented statements into the discussion section. Finally, to guide the reader through the analyses more clearly, we now end each subsection with a brief summary that highlights the key takeaway.

The Discussion effectively highlights the conceptual contribution of the N3 framework and situates it within the broader goals of precision medicine. The authors clearly articulate how N3 advances beyond population-average models and link their findings to practical implications in neurodegenerative disease research and early diagnosis. To improve, the Discussion should streamline its messaging and trim details already covered in previous sections, allowing greater focus on interpretation and conceptual insight. The authors should further emphasize the theoretical and clinical significance of their findings — for example, how N3 might reshape normative modeling practices or inform individualized treatment strategies. While the study's limitations are acknowledged, their practical impact on smaller clinical studies and potential strategies for mitigation deserve deeper exploration. The section would also benefit from a clearer synthesis of how the results advance understanding in the field and from outlining concrete next steps for research and clinical translation.

We thank the reviewer for the constructive assessment of our discussion section. In response, we have substantially revised and streamlined the messaging, e.g. by removing content that was redundant with earlier sections. We now more explicitly articulate the theoretical significance of N^3 and clarify how this perspective can reshape normative modeling practices beyond population-average approaches. We also expanded the clinical perspective and have deepened our analysis of the practical impact on smaller clinical studies. We added a concise synthesis paragraph that highlights how our results advance understanding in the field and now explicitly outline key next steps for research and clinical translation, including extending N^3 to high-dimensional imaging representations, integrating genetic and environmental risk factors, and systematically evaluating performance in routine clinical settings.

The Conclusion succinctly summarizes the core contribution of the study and reiterates its potential relevance to personalized medicine. It effectively reinforces the translational promise of the N3 framework across medical domains. The authors could more explicitly link the closing statements to the key empirical findings and highlight what the study concretely adds to existing knowledge. They should also clarify the specific next steps for validation or clinical implementation, as the current phrasing remains largely aspirational. Finally, tightening the language to avoid repetition (e.g., “personalized healthcare” and “personalized patient care”) would improve clarity and impact.

We thank the reviewer for this constructive feedback on our conclusion. In response, we have fully revised the concluding paragraph to more clearly situate our findings in a broader conceptual and clinical context, emphasizing how N³ reframes the definition of normativity and supports our collective endeavor of realizing individualized patient care.

Methods

-Major Concerns:

Conceptual clarity and reproducibility: The description of the N3 algorithm is mathematically comprehensive but would benefit from clearer organization. Presenting the conceptual framework separately from the implementation details (e.g., the neuroimaging case) would make the methods easier to navigate and improve reproducibility.

We thank the reviewer for this constructive suggestion. We have substantially reorganized the Methods section to clearly delineate the conceptual framework of the N³ algorithm from its specific implementation in our neuroimaging application. The algorithmic description now follows a hierarchical structure that progresses from general principles to domain-specific choices, enhancing both conceptual clarity and reproducibility across different application domains.

Equation (2) – Density estimation: The formula does not align with standard local density estimation practices and lacks normalization by dimensionality or the number of neighbors (k). A justification for this formulation should be provided.

We appreciate the reviewer's attention to this detail. The formulation has been corrected to include explicit normalization by the number of neighbors k, now reading:

$$\lambda(q_i, c) = (\frac{1}{k}\sum_{j=1}^{k}\|x_j - q\|)^{-1}$$

Equation (5) – Exponentiated Weibull distribution: The distribution's mathematical form appears incomplete (missing a scale parameter), and parameter estimation procedures are not described. The rationale for choosing this distribution for λ′ values should be clarified.

We have expanded our exposition of the exponentiated Weibull distribution choice. The Weibull distribution provides flexibility in accommodating diverse distributional shapes, including left-skewed, right-skewed, symmetric, and multimodal configuration, in order to accommodate any heterogeneous density patterns across different demographic subgroups. Parameters are estimated via maximum likelihood estimation using the scipy.stats.exponweib implementation. The complete probability density function is:

$$f(x;\alpha,\beta,\gamma) = \frac{\gamma\alpha}{\beta}\left(\frac{x}{\beta}\right)^{\alpha-1}\exp\left[-\left(\frac{x}{\beta}\right)^{\alpha}\right]\left\{1-\exp\left[-\left(\frac{x}{\beta}\right)^{\alpha}\right]\right\}^{\gamma-1}$$

We have added this specification to the manuscript along with a brief justification of its superior fit compared to standard parametric alternatives.

Equation (6) – Signed likelihood rule: The conditional “if x < 1” seems arbitrary after normalization and should instead compare against the median value to correctly determine direction of deviation.

We have revised the conditional statement to properly reference the subgroup-specific median.

$$\ell^*(q,c) = \begin{cases} -\ell(q,c) & \text{if } \lambda(q,c) < \text{median}(\Lambda_c) \\ \ell(q,c) & \text{otherwise} \end{cases}$$

Parameter k selection: The fixed choice of k = min(round(0.1 × n), 15) is arbitrary and not empirically justified. A sensitivity analysis or rationale for this threshold should be included.

We thank the reviewer for outlining this shortcoming. We have added a sensitivity analysis and repeated the statistical and machine learning analyses for $k \in [5, 35]$. The sensitivity analysis reveals overall robustness of the N³ biomarker, with disease-specific optimal configurations and two general observable trends. First, $k > 20$ tends to decrease performance and second, with increasing k, the trade-off between recall and precision is shifted towards higher recall. We have added the figures below as well as a comprehensive result section in the Supplementary Materials.

Supplementary Results: *To establish the impact of the hyperparameter k in our application of the N³ framework, we have added a sensitivity analysis and repeated the statistical and machine learning analyses for $k \in [5, 35]$.*

The sensitivity analysis demonstrates robust N³ performance across the tested parameter range, with performance metrics exhibiting minimal variation for changes in $k < 15$. We see two general observable trends: First, values for $k > 20$ tend to decrease performance and second, with increasing k, the trade-off between recall and precision is shifted towards higher recall.

The sensitivity analysis revealed disease-specific optimal configurations in group-level analyses. Statistical discrimination peaks at k=5 for AD (η_2=0.299), k=15 for MCI (η_2=0.067) and k=15 for FTD (η_2=0.376), reflecting differential spatial scales of pathological alterations. For AD, statistical discrimination declines monotonically with increasing k, suggesting that fine-grained local density estimation optimally captures AD-related brain alterations. Conversely, FTD exhibits peak discrimination at k=15, indicating that moderately larger neighborhoods better characterize frontotemporal pathology patterns. MCI shows relatively stable but modest effect sizes across $k \in [10,20]$.

Classification performance too exhibited disease-dependent optimal k values. AD achieves peak balanced accuracy at k=15 (0.792) with a high F1-score (0.631), representing a good trade-off between precision and recall. FTD classification shows a precision-recall trade-off, while k=5 maximizes recall (0.805), k=25 yields highest precision (0.880) and balanced accuracy (0.786). MCI demonstrates consistent but modest classification performance (balanced accuracy 0.610–0.620), with marginal advantage at k=15 (0.620).

The discordance between optimal k for statistical discrimination versus classification, particularly evident in FTD and MCI, reflects the distinction between group-level distribution shifts and individual-level decision boundary optimization.

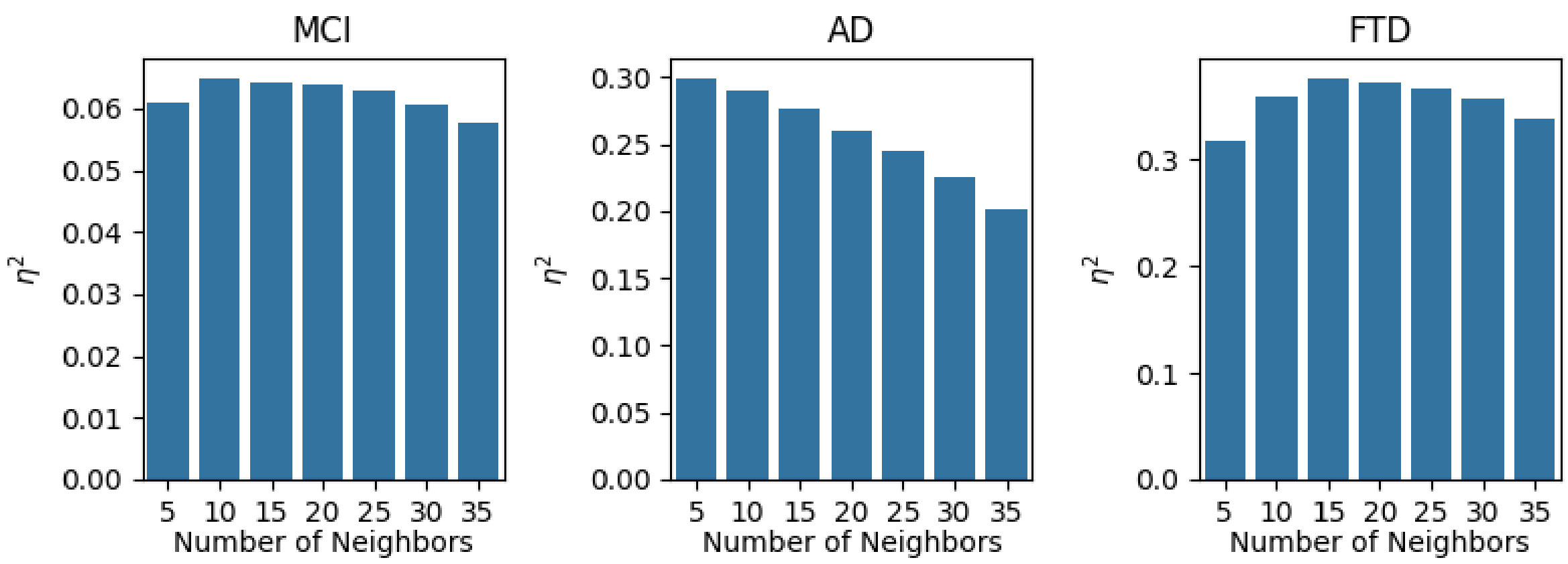

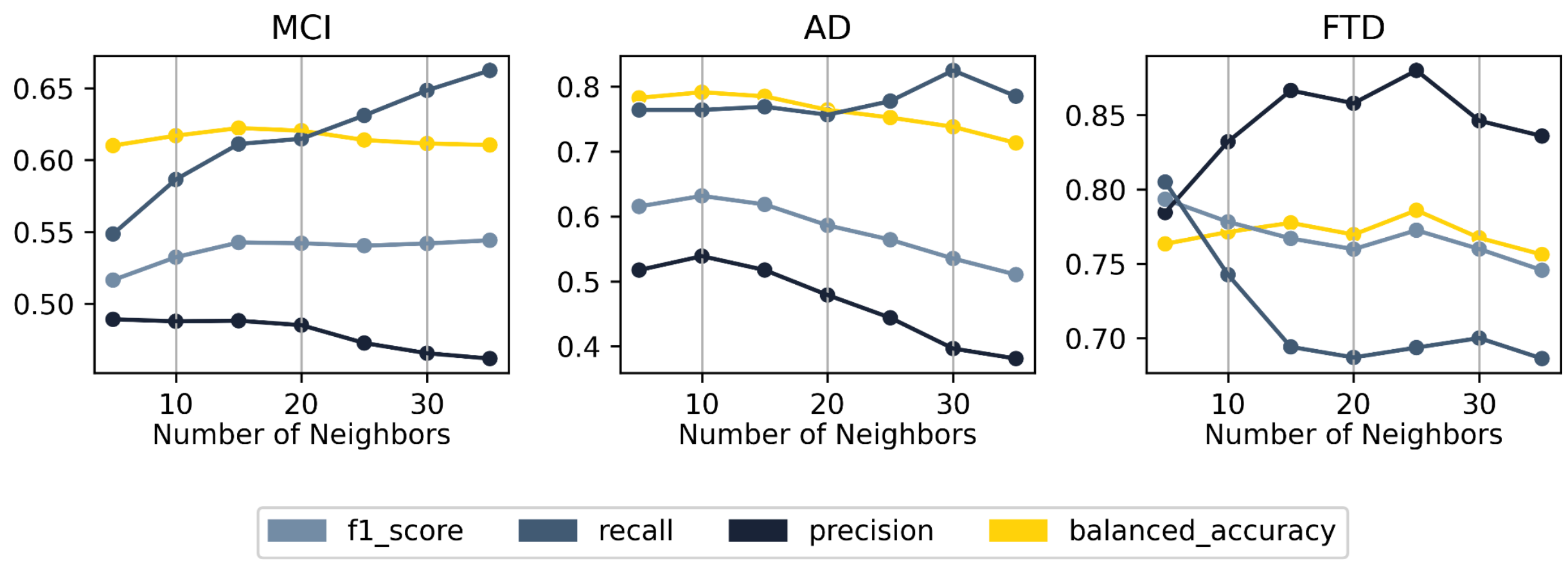

Meta-normativity layer (Eq. 9): The second-layer estimation step is conceptually under-specified. The authors should clarify what distance metric and normalization procedures are used when comparing normativity profiles and how independence between layers is maintained.

We have substantially clarified the second-layer of normativity estimation. The second layer applies the identical local density estimation framework described in Methods section 5.1.1, but operates in the g-dimensional normativity profile space rather than the original m-dimensional feature space. Specifically, each individual's normativity profile φ(q) replaces the original feature vector as input in an additional local density estimation procedure. Thereby, we further contextualize the measurements of the individual against examples from the reference population.

Methods 5.1.2: In the next step, we aggregate normativity estimations from several subgroups into a vector, which we call normativity profile and which depicts an individual's normativity measured from multiple meaningful angles or viewpoints. To synthesize the multidimensional information entailed in an individual normativity profile into a single, actionable metric, we repeat the local density estimation approach outlined above. In a second layer of normativity estimation, we use the normativity profile φ as input data and repeat the local sample density estimation approach outlined above. Now, the local density estimation is based on the g values of φ, computed in the normativity profile space. Thereby, we measure the commonness of a normativity profile in relation to other normativity profiles seen in a particular reference population. This can either be done globally (on all normativity profiles of the sample), or again in subgroups. The output of this meta-normativity estimation is the return value of the N³ algorithm, what we call the N³ biomarker.

-Minor Concerns:

Inconsistent notation (e.g., alternating between q, x, and p for samples, and between ϕ and φ for normativity profiles).

We thank the reviewer for outlining this source of confusion and have substantially revised and streamlined the algorithm description, including an optimized notation. We now consistently use q to denote query samples and φ (phi) to denote normativity profiles, and we have added a tabular overview providing a variable glossary for reader reference.

Typographical error ("medium" should read "median").
Corrected. We thank the reviewer for catching this.

The term "control group" is used variably to mean both a demographic subgroup and a reference sample — clearer terminology would help.
We have replaced "control group" with "subgroup" throughout to avoid confusion and to more accurately reflect the demographic stratification approach.

Merging adjacent age bins in Section 5.1.5 could introduce bias or information leakage across strata and should be statistically justified.

We thank the reviewer for pointing out this potential source of bias. In our application, we use annual age stratification (ages 22–72) distinctively for both sexes. To ensure stable local density estimation while preserving age specificity, we implement a conservative merging protocol for underrepresented age strata. Specifically, bins falling below the sex-specific median sample size are augmented by pooling with immediately adjacent age groups (±1 year maximum). The merging is strictly local (maximum span of 3 years) and respects the continuous, smooth trajectory of neuroanatomical aging, wherein structural brain volumes exhibit high autocorrelation across adjacent years. Moreover, increased intra-stratum heterogeneity elevates density estimate variance, potentially attenuating rather than inflating sensitivity to true deviations We believe this conservative, data-driven approach appropriately balances the competing demands of age specificity and stability in density estimation.

The inference-phase description (lines 370–379) lacks detail on how stored parameters from the second layer are reused for new samples.
We thank the reviewer for pointing out this inconsistency. We have substantially revised the whole section for a clearer understanding, outlining the training workflow, the stored parameters and how the models are applied during the inference phase.

Figures and Tables
We thank the reviewer for outlining the shortcomings in our figures and tables, enabling us to improve the clarity of the presented information.

- Figure 1 and Figure 2: Including both figures in the Introduction is unconventional; they primarily describe the algorithmic framework and would be better placed in the Methods or Supplementary Material. Figure 1's depiction of "normativity profiles" largely repeats the text; the visual should emphasize workflow, data flow, or hierarchical structure to add distinct value.
Figure 2's example profiles (three individuals) require labeled axes and a clear legend clarifying what "alignment" represents and how to interpret deviations.

We have relocated Figure 1 to the Methods section and revised both the figure and its legend to better emphasize the workflow and data flow. Moreover, we have moved Figure 2 to the Results section and updated its axes, legend, and description to clarify what the profiles represent

Fig workflow: The N³ framework analyzes the local sample density around individual measurements in strategically chosen subgroups of a normative reference dataset. This strategy accommodates multiple, equally viable health states in the population and promotes the detection of nuanced irregularities that may escape broader comparative models. Moreover, by evaluating the local sample density in multiple strategically chosen subgroups, the N³ framework provides a multifaceted contextualization of medical observations. We call the resulting vector a normativity profile. The information contained in the normativity profile is converted into a singular, actionable metric, by re-applying local density estimation. The resulting metric expresses how common a normativity profile is for a particular demographic or clinical subgroup. It can be adapted to a variety of clinical inquiries, such as how common is this profile amongst young women, treatment resistant patients or genetic predispositions.

Fig. 2 Example profiles: "For illustration, we show the normativity profiles of three individuals of the training sample. The N³ framework compares an individual's brain structural features (global volumes of gray matter, white matter, white matter hyperintensities, cerebrospinal fluid and total intracranial volume) to those seen in different age groups along the age continuum. The normativity profile shows the local sample density in different age groups, i.e. how common similar volumetric configurations appear along the aging continuum (blue) as well as the individual's chronological age (green). (a) An individual's data shows higher local sample density in younger age groups, indicating that the volumetric configuration is more common within younger age groups and less common within same-aged individuals (b) An individual's volumetric configuration appears more common within older age groups, indicating premature aging effects. (c) A brain structure appears most common within its own age group and deprecating occurrence within other age groups."

- Figure 3: The top panel presents effect sizes (η^2), but the axes, units, and color scheme are unclear — axis labels and a complete legend should be added. The bottom panel includes multiple metrics (balanced accuracy, F1-score, recall, precision) that make the plot crowded; highlighting the main metric or separating panels could improve readability. Captions should remain descriptive — current phrasing mixes figure content with interpretive commentary (e.g., "larger explained variance").

We have separated the figure into a statistical part and a machine-learning part, added clear axis labels and a complete legend, and revised the captions to remain descriptive without interpretive commentary.

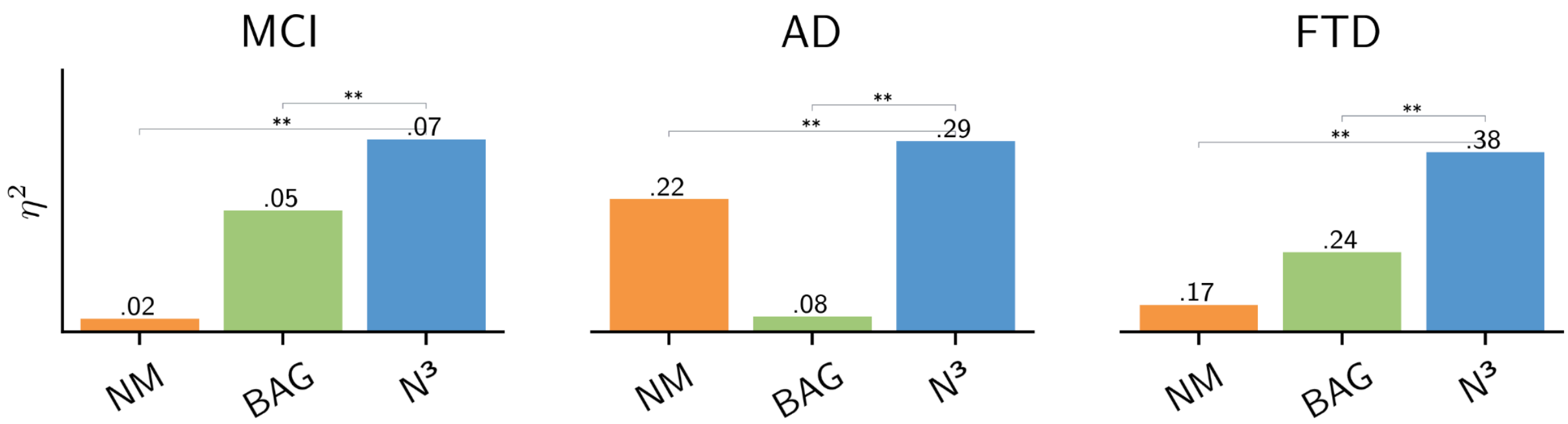

*Fig. 3 Statistics: Statistical effect sizes (partial eta squared - η^2) are given for the different biomarkers (N³ - our approach, NM - Normative Modeling outlier score, and the BAG - Brain Age Gap). We evaluate each approach's ability to differentiate between controls and diseased individuals using neurodegenerative diseases as representative models diseases, in particular Mild Cognitive Impairment (MCI), Alzheimer's Disease (AD), and Frontotemporal Dementia (FTD). Post-hoc comparisons of the effect sizes revealed larger explained variance of our N³ marker in all neurodegenerative conditions. Significance in the differences was confirmed through permutation testing using 1000 random permutations and is indicated above (** $p<.001$).*

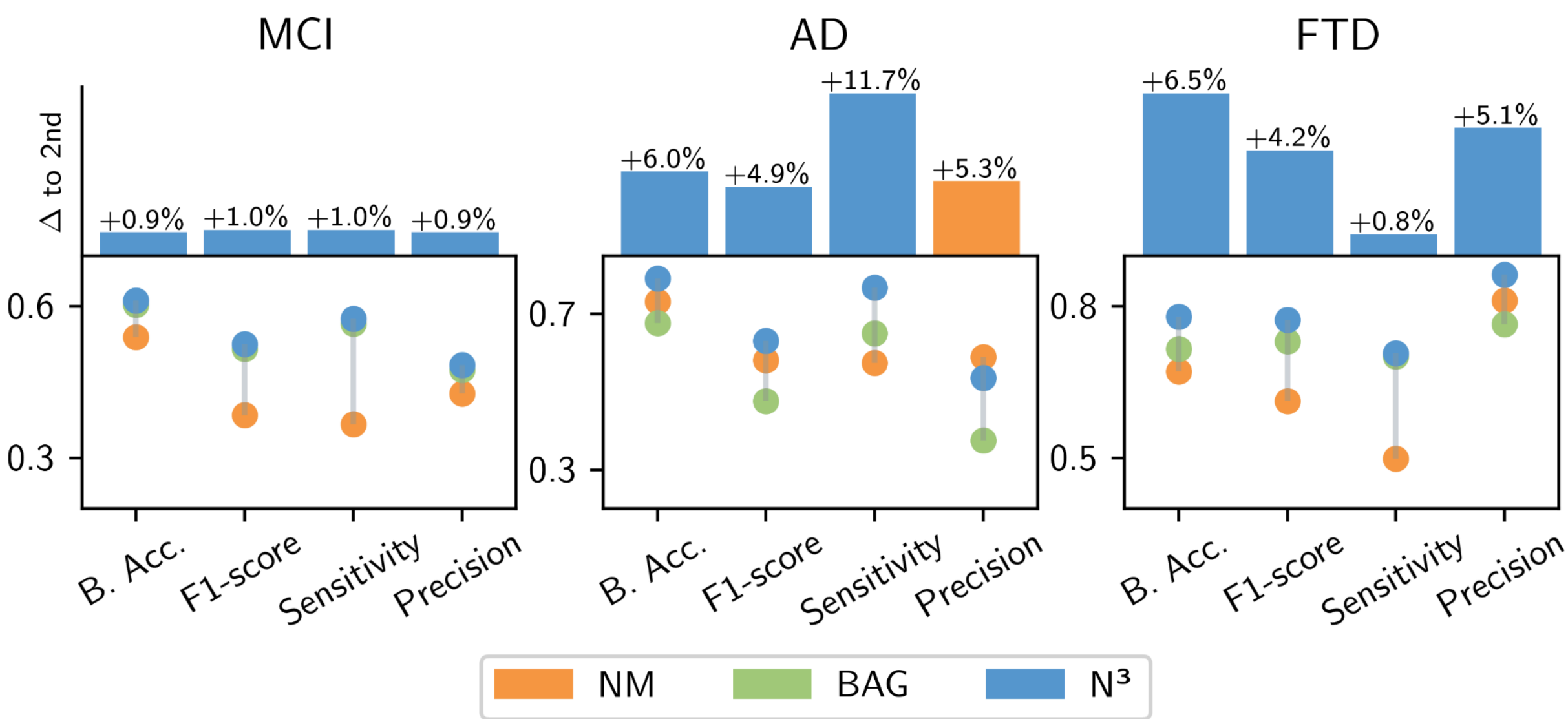

Fig. 3 Machine Learning: We use machine learning to evaluate the expressiveness of each biomarker on a single-subject level (N³ - our approach, NM - Normative Modeling outlier score, and the BAG - Brain Age Gap). We evaluate each approach's ability to differentiate between cognitively unimpaired and diseased individuals using neurodegenerative diseases as representative models diseases, in particular Mild Cognitive Impairment (MCI), Alzheimer's Disease (AD), and Frontotemporal Dementia (FTD). The N³ marker demonstrated increased accuracy in predicting the occurrence of pathological norm deviations. We show the different marker's performance metrics [balanced accuracy (B.Acc), F1-Score, Recall and Precision] and the performance advantage, .i.e. the delta, of the best biomarker in relation to the second best biomarker in percentage (Δ to 2nd).

- Figure 4: The ICC analysis is relevant, but subplots (a–c) should be explicitly labeled and referenced in the caption for clarity. Replace subjective phrasing such as “converges to excellent levels” with quantitative language (e.g., “ICC ≥ 0.9 for n ≥ 300”).

We have explicitly labeled the subplots in the figure and caption and updated the descriptions of ICC values in line with the suggestion.

- Table 1:The table effectively summarizes statistical and machine-learning results but is dense; splitting it into two (statistical vs. predictive metrics) or moving detailed values to Supplementary Material would enhance readability. Ensure consistent bolding of best-performing results and define all abbreviations (NM-S, NM-C, BAG, N^3) directly in the caption.

We have split the original table into separate tables for statistical and predictive results, ensured consistent highlighting of best-performing entries, and added definitions of all abbreviations directly in the captions.

- Table 2: The dataset summary is valuable but could be formatted for easier reading (aligned columns, consistent decimal places). Add brief notes on inclusion/exclusion criteria and clarify whether participants contributed multiple scans to improve transparency.

We have fixed the alignment issues in Table 2 and updated the caption to provide the requested additional information.

Response Letter

We sincerely thank all reviewers for the thorough and constructive review of our revised manuscript. We are grateful for the time invested in evaluating our research. Below, we have addressed all remaining concerns and provide our response to the specific remarks from each reviewer.

Reviewer #1 (Remarks to the Author):

The authors have thoroughly addressed the points raised in the initial review. Specifically, the additional clarifications and formula corrections in the Methodology and Results section have strengthened the manuscript's findings. I am satisfied with the current version and believe it now meets the standards for publication. I recommend acceptance.

We are grateful for the reviewer's initial feedback which identified critical areas where the methodology and results required greater clarity and rigor and appreciate their recommendation for acceptance.

Reviewer #2 (Remarks to the Author):

I appreciate the authors' efforts in clarifying the mathematical framework of the N3 algorithm. The revised methods section has significantly improved the explanation of this approach.

While the model remains limited by its reliance on global volumetric measures rather than local spatial patterns, the authors have successfully argued that N3 is intended to capture how multivariate volumetric patterns of a particular individual correlate with those in different aging subgroups, which would be missed by univariate or standard normative strategies. I also thank the authors for incorporating this limitation in the discussion.

We would like to thank the reviewer again for their thoughtful engagement with our N^3 framework and the constructive feedback on the scope and limitations of the approach. In the revised manuscript, we have further clarified the current work as proof-of-concept validation (see below) and strengthened the relevance of future extensions to whole-brain representations.

Reviewer #4 (Remarks to the Author):

I thank the Editor for the opportunity to evaluate the revised version of this manuscript and the accompanying rebuttal letter.

The authors have substantially revised the manuscript and addressed many of the concerns raised during the previous review round. The revised version is considerably clearer in its

methodological presentation and in the conceptual framing of the proposed Nearest Neighbor Normativity (N^3) framework.

Overall, I find the work interesting and potentially relevant for the neuroimaging and computational neuroscience communities. The manuscript addresses an important and timely issue, namely the limitations of population-average approaches for modeling heterogeneous brain aging trajectories and individualized norm deviations. The proposed framework introduces a contextualized and density-based perspective on normativity assessment that differs conceptually from both classical normative modeling and brain-age paradigms.

One of the main strengths of the manuscript is that it reframes normativity not as a deviation from a single average trajectory, but as a structured space of plausible configurations. I believe this conceptual perspective is novel and may stimulate further discussion in the field regarding the role of inter-individual variability in neuroimaging biomarkers and personalized medicine approaches.

The authors have also improved several aspects of the manuscript in response to the reviewers' comments, including:

-clarification of the N^3 computation and notation,
-better explanation of the machine learning evaluation setup,
-expanded discussion of biological interpretability,
-additional analyses for normative modeling aggregation strategies,
-and clarification of the BAG bias correction procedure.

The manuscript is now substantially more understandable and methodologically transparent than the previous version.

Nevertheless, I believe some important limitations should still be more explicitly acknowledged and discussed.

First, while the manuscript conceptually emphasizes "multiple normative prototypes" and a "landscape of plausible configurations", the current implementation remains largely based on demographic subgrouping strategies (primarily age- and sex-matched local neighborhoods). As currently formulated, the plurality of normative states appears to arise mainly from localized demographic contextualization rather than from the identification of biologically distinct latent trajectories. I therefore encourage the authors to slightly moderate some conceptual claims and clarify this distinction more explicitly.

Thank you for this constructive critique. We appreciate the opportunity to edit our description of the algorithmic approach for clarification. The plurality of normative states does not arise from the demographic stratification itself, but from the application of local density estimation techniques within demographic subgroups. Within each demographic subgroup, we identify peaks of local sample density in the multi-dimensional biomarker space. Each density peak represents a distinct biological configuration that co-exists with other configurations within a given age and sex group, thereby capturing heterogeneous biological configurations. We

agree with the reviewer that this distinction is critical and have clarified it throughout the Introduction and Methods sections.

***Introduction**: By relying on context-enriched assessments, the N³ framework is designed to improve the understanding of variance in-between individual brain structures within the natural diversity seen along the aging continuum. Specifically, the N³ framework employs local density estimation within demographic subgroups, which allows multiple plausible biological configurations to co-exist within the context of an age- and sex-matched population. The local sample density estimation captures natural heterogeneity while the anchoring to demographic subgroups ensures that comparisons occur among biologically similar individuals and remain clinically interpretable.*

***Methods**: Within these subgroups, N³ quantifies normativity via local sample density estimation, determining how frequently similar value combinations occur within local neighborhoods of a measurement space. Multiple density peaks can exist within a single neighborhood, so that, for example, an age and sex group may contain several plausible normative patterns. Rather than averaging across these configurations, N³ retains their plurality, allowing each individual's configuration to be compared against the most appropriate local reference.*

Second, although the framework is presented as broadly applicable to complex neurobiological representations, the current validation relies on a relatively low-dimensional set of global volumetric measures (GM, WM, WMH, CSF, TIV). The authors appropriately acknowledge this limitation in the revised Discussion. However, I believe the manuscript would benefit from a clearer statement that the current study primarily demonstrates the conceptual feasibility of the framework rather than a definitive validation in genuinely high-dimensional neuroimaging settings.

We agree that the current validation represents proof-of-concept in relatively low-dimensional space. Beyond the Discussion, we have now additionally addressed this in the Introduction and Methods sections.

***Introduction**: We use a set of global volumetric biomarkers (gray matter, white matter, white matter hyperintensities, cerebrospinal fluid, and total intracranial volume) to demonstrate conceptual feasibility and benchmark the resulting N³ biomarker relative to conventional neuroimaging biomarker approaches, including conventional normative modeling and Brain Age models.*

***Methods**: The local density estimation is applied across five volumetric summary measures (gray matter, white matter, white matter hyperintensities, cerebrospinal fluid, total intracranial volume) to identify normative configurations. While this dimensional reduction enables a robust prototyping of the framework and facilitates validation across large heterogeneous cohorts, the framework is not inherently limited to these measures, and extensions to whole-brain representations are considered in the Discussion..*

Third, while the methodological description has improved substantially, some details regarding subgroup construction and overlap remain somewhat difficult to fully reconstruct. Additional clarification regarding subgroup overlap, neighborhood definition, subgroup size

selection, and statistical dependence between subgroups would further improve reproducibility.

We thank the reviewer for pointing out this shortcoming. We have substantially expanded the Methods section and explicitly describe the initial stratification (1-year age bands by sex) within the five-dimensional biomarker space and the joining procedure (merging of underrepresented groups with lower and upper neighbors). With regard to statistical dependence: Overlapping neighborhoods do not affect statistical group comparisons (e.g., controls vs. disease) because N^3 assessment follows a two-step structure: First, individual normativity profiles are computed across the entire continuum irrespective of true chronological age. To generate a normativity profile, each configuration is evaluated within all neighborhoods across all same-sex age groups. If present, each normativity profile would therefore exhibit the same systematic bias from between-group dependencies across all age groups. However, the normativity profile itself is evaluated only within same-aged peers for statistical testing, with no merging of adjacent age groups in this second evaluation step. Thus, the overlapping neighborhood design is part of the feature generation process only, and does not propagate to the statistical tests comparing disease groups. We apologize for the lack of clarity in the description. The two-step evaluation is now explicitly detailed in the Methods section, too.

Methods: *We stratify the training sample by sex and age, resulting in a total of 100 demographic groups containing same-aged females or males (22 to 72 years), respectively. To mitigate unequal sample sizes across age groups (due to cohort age distribution), we implemented a joining procedure where underrepresented age groups (defined as having n < median sample size per sex) were merged with the adjacent 1-year age band. The merging procedure first joined the immediately lower neighboring age group, then, if still underrepresented, joining the upper neighboring group. As adjacent age groups share similar brain structural characteristics, this strategy supports smooth normative transitions across the aging spectrum and ensures comparable variability in the neighborhoods across the aging continuum.*

Within each demographic neighborhood, the neighborhood in the five-dimensional biomarker space was characterized by local sample density computed using k-nearest neighbors, where $k = \min(0.1*N, 15)$. The 10% rule ensures that k scales with neighborhood size while the upper bound of $k=15$ prevents excessively broad comparisons that would override fine-grained local density structure.

The N^3 algorithm proceeds in two steps. First, for each individual, we compute local sample density estimates within each age-year neighborhood across the entire continuum (ages 22–72), generating 50 local density values (one per year per sex). These are combined into a normativity profile that captures the individual's normativity across the entire aging spectrum. Balancing the variability in the neighborhoods across the aging continuum is relevant to this first level of local sample density estimation, as all estimates are then jointly evaluated in the resulting normativity profile. In contrast, in the second layer of local density estimation, the individual's normativity profile is evaluated within the same-aged and same-sex peer group only, deriving the final N^3 biomarker. This two-step structure ensures that individuals are first contextualized within the broader aging continuum, then assessed relative to exact age- and sex-matched peers.

Regarding statistical validity, the analyses appear generally appropriate and carefully conducted. I particularly appreciate the additional clarification regarding the BAG bias correction strategy and the inclusion of multiple normative modeling aggregation approaches. The comparative evaluation across statistical effect sizes and single-feature classification analyses is coherent with the stated goals of the study.

Overall, I believe this revised manuscript represents a thoughtful and conceptually interesting contribution to the discussion surrounding personalized normative assessment in neuroimaging. While some conceptual claims may still benefit from slightly more cautious framing, I believe the manuscript has been substantially strengthened through revision and is close to being suitable for publication after relatively minor clarifications.
