# Supplementary material for: Contextualizing biological heterogeneity in brain structural normativity assessments across the aging continuum with the nearest neighbor normativity framework
Source: Commun Med (Lond). 2026 Nov 13;6:587. doi: 10.1038/s43856-026-01903-0 (PMC13768337; doi:10.1038/s43856-026-01903-0)
Supplement: Supplementary file 2 [file 43856_2026_1903_MOESM2_ESM.pdf]

Contextualizing Biological Heterogeneity in Brain Structural Normativity Assessments Across the Aging Continuum with the Nearest Neighbor Normativity Framework

Leenings et al.

Supplementary Information

Sensitivity Analysis

To establish the impact of the hyperparameter k in our application of the $\mathbf{N^3}$ framework, we have added a sensitivity analysis and repeated the statistical and machine learning analyses for $k \in [5, 35]$.

The sensitivity analysis demonstrates robust $\mathbf{N^3}$ performance across the tested parameter range, with performance metrics exhibiting minimal variation for $\Delta k < 15$. We see two general observable trends: First, values for $k > 20$ tend to decrease performance and second, with increasing k, the trade-off between recall and precision is shifted towards higher recall.

The sensitivity analysis revealed disease-specific optimal configurations in group-level analyses (see Table S1 and Fig. S1). Statistical discrimination peaks at k=5 for AD (η^2=0.299), k=15 for MCI (η^2=0.067) and FTD (η^2=0.376), reflecting differential spatial scales of pathological alterations. For AD, statistical discrimination declines monotonically with increasing k, suggesting that fine-grained local density estimation optimally captures AD-related brain alterations. Conversely, FTD exhibits peak discrimination at k=15, indicating that moderately larger neighborhoods better characterize frontotemporal pathology patterns. MCI shows relatively stable but modest effect sizes across $k \in [10, 20]$ ($\eta^2$0.064).

Classification performance too exhibited disease-dependent optimal k values (see Table S2 and Fig. S2). AD achieves peak balanced accuracy at $k \approx 10-15$ (0.791) with a high F1-score (0.631), representing a good trade-off between precision and recall. FTD classification shows a precision-recall trade-off, while $k = 5$ maximizes recall (0.805), $k = 25$ yields highest precision (0.880) and balanced accuracy (0.786). MCI demonstrates consistent but modest classification performance (balanced accuracy 0.610–0.620), with marginal advantage at k=15 (0.620).

The discordance between optimal k for statistical discrimination versus classification, particularly evident in FTD and MCI, reflects the distinction between group-level distribution shifts and individual-level decision boundary optimization.

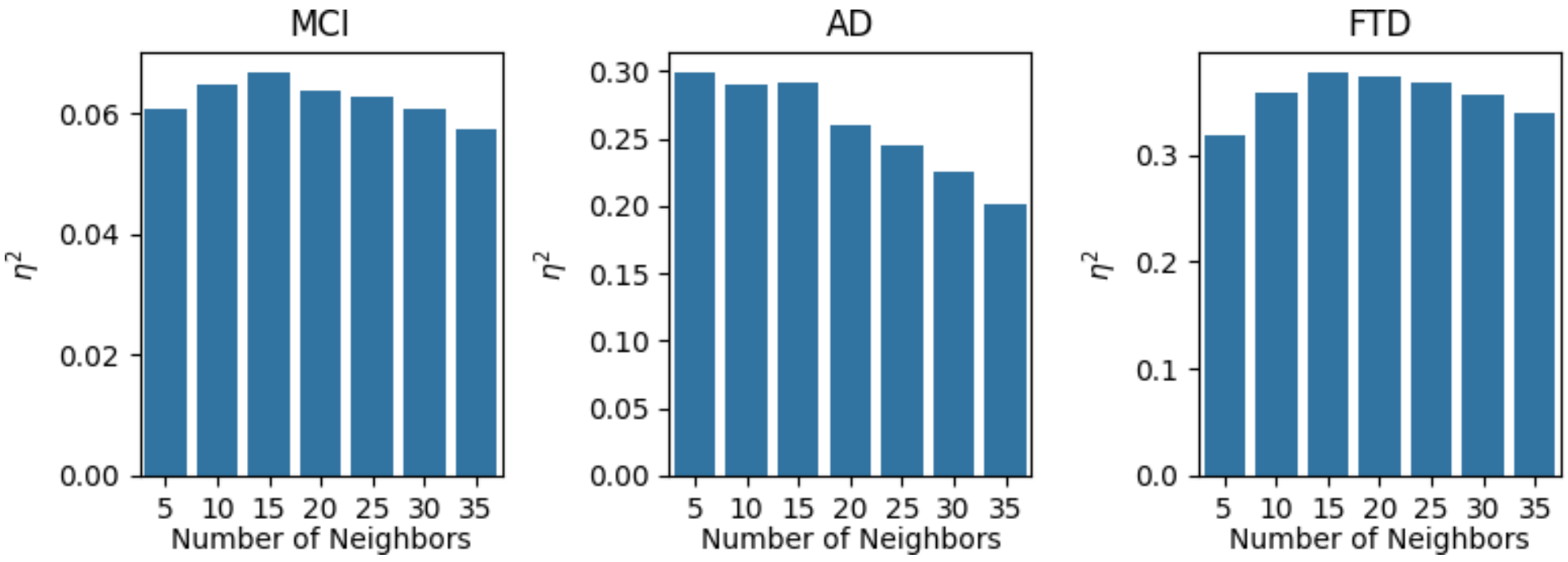

Fig. S1 Statistical effect sizes for different values of the nearest neighbor parameter k. Effect size, partial eta-squared (η^2), is depicted, comparing the discriminative power of the N^3 biomarker across neurodegenerative diseases for different values of the nearest neighbor parameter k (MCI - Mild Cognitive Impairment, AD - Alzheimer's Disease, FTD - Frontotemporal Dementia).

Table S1 Statistical sensitivity analysis of nearest neighbor parameter k. Effect of the nearest neighbor parameter k on the discriminative power of the N^3 biomarker across neurodegenerative diseases (MCI - Mild Cognitive Impairment, AD - Alzheimer's Disease, FTD - Frontotemporal Dementia). F-statistics and partial eta-squared (η^2) effect sizes comparing patient groups to cognitively unimpaired individuals. Bold indicates optimal results per disease. All p<0.001.

	**MCI**		**AD**		**FTD**	
**k**	**F**	η^2	**F**	η^2	**F**	η^2
5	F(1,4565)=296	0.061	F(1,3709)=**1,577**	**0.299**	F(1,580)=269	0.318
10	F(1,4565)=312	0.065	F(1,3709)=1,510	0.290	F(1,580)=321	0.358
15	F(1,4565)=**313**	**0.067**	F(1,3709)=1,412	0.292	F(1,580)=**346**	**0.376**
20	F(1,4565)=311	0.064	F(1,3709)=1,302	0.260	F(1,580)=342	0.373
25	F(1,4565)=306	0.063	F(1,3709)=1,198	0.244	F(1,580)=335	0.367
30	F(1,4565)=295	0.061	F(1,3709)=1,074	0.225	F(1,580)=319	0.356
35	F(1,4565)=279	0.058	F(1,3709)=932	0.201	F(1,580)=296	0.339

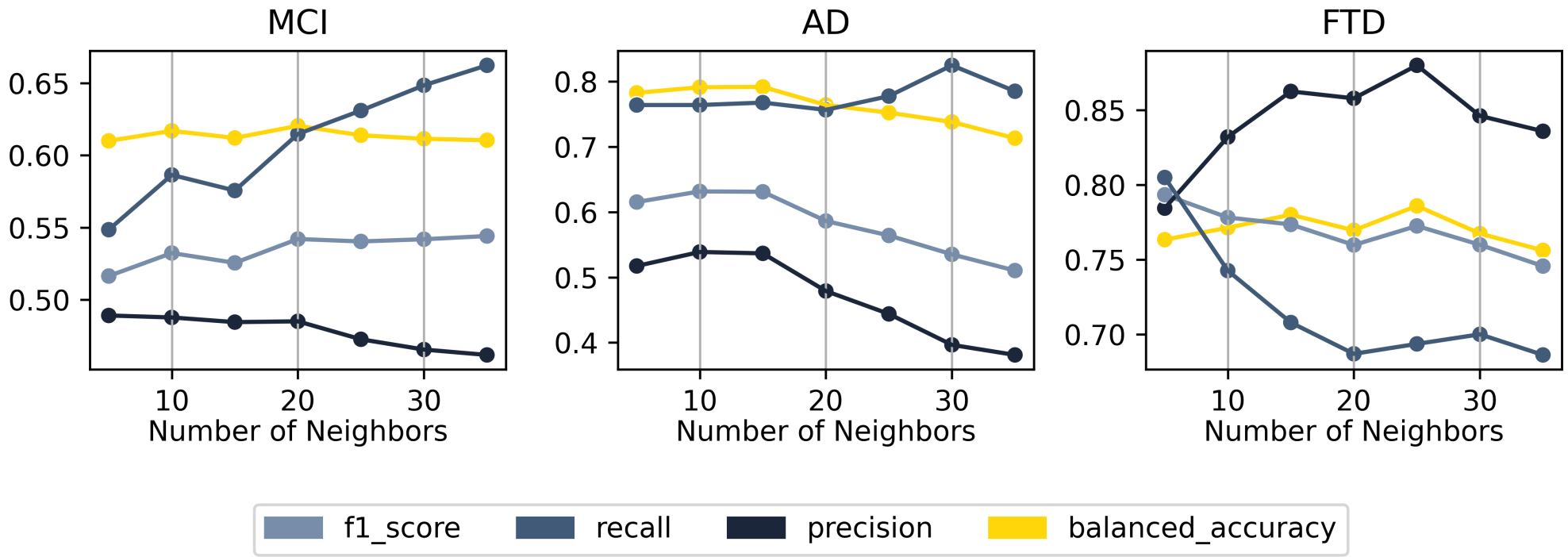

Fig. S2 Machine Learning performance for different values of the nearest neighbor parameter k. Performance metrics for the N^3 biomarker for different values of the nearest neighbor parameter k in the classification of cognitively unimpaired individuals from those with neurodegenerative diseases (MCI - Mild Cognitive Impairment, AD - Alzheimer's Disease, FTD - Frontotemporal Dementia).

Table S2 Classification performance for different values for the nearest neighbor parameter k. Bold values indicate best performance per disease and metric.MCI - Mild Cognitive Impairment, AD - Alzheimer's Disease, FTD - Frontotemporal Dementia; Bal. Acc. - Balanced Accuracy. Bold indicates best performance per disease.

Disease	k	Bal. Acc.	Recall	Precision	F1 Score
MCI	5	0.610	0.548	**0.489**	0.516
MCI	10	0.617	0.586	0.488	0.532
MCI	15	0.612	0.576	0.484	0.526
MCI	20	**0.620**	0.615	0.485	0.542
MCI	25	0.614	0.631	0.473	0.540
MCI	30	0.611	0.648	0.465	0.542
MCI	35	0.610	**0.662**	0.462	**0.544**
AD	5	0.783	0.764	0.517	0.615
AD	10	0.791	0.764	**0.539**	**0.632**
AD	15	**0.792**	0.768	0.537	0.631
AD	20	0.764	0.757	0.479	0.586
AD	25	0.752	0.778	0.444	0.564
AD	30	0.738	**0.825**	0.396	0.535
AD	35	0.713	0.785	0.381	0.510
FTD	5	0.763	**0.805**	0.784	**0.793**
FTD	10	0.771	0.743	0.832	0.778
FTD	15	0.780	0.708	0.863	0.773
FTD	20	0.770	0.687	0.858	0.760
FTD	25	**0.786**	0.694	**0.880**	0.773
FTD	30	0.767	0.700	0.846	0.760
FTD	35	0.756	0.686	0.836	0.746

Normative Modeling Aggregation

We evaluated different aggregation strategies of the Normative Modeling z-scores. Table S3 summarize effect sizes from group-level statistics, while Table S4 summarizes predictive performance from machine learning analyses, allowing a direct comparison of how each aggregation strategy supports disease classification. Aggregations based on the overall count of outliers and the absolute sum of deviations yielded consistently larger effect sizes in the statistical analyses, whereas averaging and the UMAP-based aggregation of z-scores resulted in negligible effects for MCI and AD. In contrast, machine learning classifiers benefited from UMAP-based aggregation, particularly in FTD, and from averaging in MCI, indicating that these strategies encode informative structure that can be exploited by non-linear models for case-level prediction. Overall, different diseases benefit from different aggregation strategies, while the count of outliers emerges as a robust choice for both statistical and machine learning analyses across diseases.

Table S3 Statistical discrimination of the different neurodegenerative diseases for different aggregation strategies of the normative modeling z-scores.

	**MCI**		**AD**		**FTD**	
**Aggregation**	**F**	η^2	**F**	η^2	**F**	η^2
average	F(1,4565)=1.45	0.0003	F(1,3709)=0.16	0.0000	F(1,580)=0.14	0.0002
count outliers	F(1,4565)=74	0.0159	F(1,3709)=1,073	0.223	F(1,580)=119	0.173
absolute sum	F(1,4565)=83	0.0180	F(1,3709)=969	0.207	F(1,580)=123	0.176
UMAP	F(1,4565)=0.02	0.0000	F(1,3709)=174	0.0449	F(1,580)=149	0.206

Table S4 Performance metrics of the machine learning analyses for different aggregation strategies of the normative modeling z-scores.

**Aggregation**	**B. Accuracy**	**F1-score**	**Sensitivity**	**Precision**
		**Mild Cognitive Impairment**		
UMAP	0.530 ± 0.008	0.452 ± 0.005	0.535 ± 0.019	0.392 ± 0.009
average	0.566 ± 0.014	0.415 ± 0.045	0.383 ± 0.090	0.474 ± 0.040
absolute sum	0.558 ± 0.013	0.344 ± 0.033	0.261 ± 0.032	0.507 ± 0.024
count outliers	0.539 ± 0.011	0.385 ± 0.057	0.367 ± 0.090	0.427 ± 0.030
		**Alzheimer's Disease**		
UMAP	0.641 ± 0.019	0.435 ± 0.023	0.573 ± 0.081	0.354 ± 0.013
average	0.676 ± 0.032	0.485 ± 0.048	0.518 ± 0.105	0.476 ± 0.052
absolute sum	0.726 ± 0.025	0.566 ± 0.034	0.586 ± 0.054	0.548 ± 0.017
count outliers	0.732 ± 0.023	0.582 ± 0.031	0.575 ± 0.050	0.590 ± 0.012
		**Frontotemporal Dementia**		
UMAP	0.756 ± 0.077	0.766 ± 0.069	0.729 ± 0.070	0.812 ± 0.086
average	0.685 ± 0.068	0.618 ± 0.089	0.500 ± 0.106	0.841 ± 0.114
absolute sum	0.649 ± 0.036	0.563 ± 0.051	0.438 ± 0.067	0.813 ± 0.086
count outliers	0.671 ± 0.028	0.613 ± 0.043	0.499 ± 0.063	0.812 ± 0.073

Robustness

To establish the robustness of the N^3 framework, we evaluated the stability of normativity estimates across varying reference sample sizes and compositions. We retrained N^3 models on downsampled subsets of NAKO and validated predictions on external datasets (MACS, ADNI). Stability was quantified using the Intraclass Correlation Coefficient (ICC; two-way mixed effects, absolute agreement).

Table S5: Sample Size, ICC, and Gender Data

Sample Size	ICC	Gender
39	0.5795	male
45	0.6518	male
50	0.7681	male
55	0.7854	male
39	0.6845	male
44	0.5909	male
50	0.7859	male
58	0.7759	male
58	0.7279	male
51	0.6495	male
44	0.4640	male
41	0.8102	male
62	0.6436	male
67	0.7794	male
37	0.6149	male
35	0.7846	male
65	0.7434	male
65	0.7946	male
58	0.7728	male
52	0.7565	male
45	0.7673	male
42	0.6669	male
79	0.7266	male
90	0.8367	male
101	0.8793	male
110	0.8657	male
78	0.8557	male
88	0.8133	male
101	0.8596	male
116	0.8376	male
117	0.8251	male
103	0.7672	male
89	0.6466	male
83	0.8890	male

Continued on next page

Continued from previous page

Sample Size	ICC	Gender
125	0.7140	male
135	0.8344	male
74	0.7675	male
70	0.8574	male
130	0.8554	male
130	0.8671	male
116	0.8723	male
105	0.8633	male
91	0.8560	male
84	0.7915	male
132	0.8574	male
151	0.8864	male
169	0.9175	male
184	0.9015	male
131	0.9026	male
146	0.9068	male
169	0.9506	male
193	0.8965	male
195	0.8092	male
173	0.8353	male
148	0.8765	male
138	0.9551	male
208	0.8140	male
225	0.9048	male
124	0.9005	male
117	0.8916	male
216	0.7946	male
216	0.8171	male
194	0.8972	male
175	0.9084	male
151	0.8734	male
140	0.9027	male
199	0.8472	male
227	0.8736	male
254	0.9528	male
276	0.8978	male
196	0.8626	male
220	0.8905	male
254	0.9218	male
290	0.7900	male
293	0.7870	male
259	0.7837	male
222	0.8618	male

Continued on next page

Continued from previous page

Sample Size	ICC	Gender
208	0.9432	male
313	0.8280	male
338	0.8590	male
187	0.9464	male
176	0.9425	male
325	0.8531	male
325	0.7955	male
292	0.8795	male
263	0.9224	male
227	0.8790	male
211	0.8474	male
29	0.5843	female
36	0.7147	female
42	0.6275	female
44	0.6802	female
49	0.7156	female
53	0.7577	female
56	0.6492	female
56	0.8254	female
52	0.7601	female
44	0.5542	female
42	0.8392	female
44	0.6886	female
49	0.7896	female
48	0.6702	female
54	0.7019	female
53	0.7114	female
41	0.6759	female
38	0.7152	female
63	0.7613	female
63	0.8539	female
56	0.6100	female
56	0.6252	female
61	0.7838	female
60	0.7581	female
62	0.6924	female
62	0.8582	female
57	0.7989	female
50	0.7781	female
54	0.8037	female
41	0.6966	female
28	0.5723	female
59	0.7927	female

Continued on next page

Continued from previous page

Sample Size	ICC	Gender
73	0.8342	female
85	0.8038	female
89	0.7829	female
98	0.8503	female
106	0.8962	female
113	0.7928	female
113	0.8768	female
104	0.8670	female
89	0.6958	female
84	0.8694	female
89	0.7596	female
98	0.9266	female
96	0.8500	female
109	0.8018	female
106	0.8647	female
82	0.7825	female
76	0.8624	female
127	0.8439	female
126	0.9226	female
113	0.7748	female
113	0.8040	female
123	0.7582	female
121	0.8483	female
124	0.7192	female
125	0.8842	female
115	0.8888	female
100	0.8608	female
109	0.8607	female
82	0.8422	female
57	0.7805	female
99	0.8739	female
122	0.8685	female
142	0.7477	female
149	0.8607	female
163	0.8619	female
176	0.9121	female
189	0.8559	female
189	0.9324	female
173	0.9185	female
149	0.8339	female
140	0.8952	female
149	0.9143	female
163	0.9481	female

Continued on next page

Continued from previous page

Sample Size	ICC	Gender
160	0.9256	female
181	0.8048	female
177	0.8680	female
138	0.8542	female
128	0.8947	female
211	0.8667	female
210	0.9343	female
189	0.8655	female
189	0.8474	female
205	0.7613	female
202	0.8853	female
207	0.7464	female
208	0.9190	female
192	0.8792	female
167	0.8998	female
183	0.8816	female
136	0.8457	female
95	0.8516	female
149	0.8923	female
183	0.9152	female
213	0.8751	female
223	0.8476	female
245	0.8317	female
265	0.9449	female
284	0.8390	female
284	0.9390	female
260	0.9602	female
224	0.7995	female
211	0.8753	female
224	0.8972	female
245	0.9301	female
240	0.9364	female
272	0.8969	female
266	0.7834	female
207	0.9092	female
192	0.9563	female
317	0.9349	female
316	0.9472	female
284	0.8906	female
284	0.8792	female
307	0.8943	female
303	0.7846	female
311	0.7907	female

Continued on next page

Continued from previous page		
**Sample Size**	**ICC**	**Gender**
312	0.9480	female
288	0.8963	female
251	0.9185	female
274	0.9019	female
205	0.8826	female
